# A pangenome analysis of ESKAPE bacteriophages: the underrepresentation may impact machine learning models

**DOI:** 10.3389/fmolb.2024.1395450

**Published:** 2024-06-21

**Authors:** Jeesu Lee, Branden Hunter, Hyunjin Shim

**Affiliations:** ^1^ Center for Biosystems and Biotech Data Science, Ghent University Global Campus, Incheon, Republic of Korea; ^2^ Department of Biology, California State University, Fresno, CA, United States

**Keywords:** ESKAPE pathogens, pangenome, protein prediction, unbalanced datasets, bacteriophages

## Abstract

Bacteriophages are the most prevalent biological entities in the biosphere. However, limitations in both medical relevance and sequencing technologies have led to a systematic underestimation of the genetic diversity within phages. This underrepresentation not only creates a significant gap in our understanding of phage roles across diverse biosystems but also introduces biases in computational models reliant on these data for training and testing. In this study, we focused on publicly available genomes of bacteriophages infecting high-priority ESKAPE pathogens to show the extent and impact of this underrepresentation. First, we demonstrate a stark underrepresentation of ESKAPE phage genomes within the public genome and protein databases. Next, a pangenome analysis of these ESKAPE phages reveals extensive sharing of core genes among phages infecting the same host. Furthermore, genome analyses and clustering highlight close nucleotide-level relationships among the ESKAPE phages, raising concerns about the limited diversity within current public databases. Lastly, we uncover a scarcity of unique lytic phages and phage proteins with antimicrobial activities against ESKAPE pathogens. This comprehensive analysis of the ESKAPE phages underscores the severity of underrepresentation and its potential implications. This lack of diversity in phage genomes may restrict the resurgence of phage therapy and cause biased outcomes in data-driven computational models due to incomplete and unbalanced biological datasets.

## Background

Bacteriophages (phages), comprising the most abundant biological entities in the biosphere, play a crucial role in various ecological and microbial systems (Suttle, 2005; Clokie et al., 2011). They contribute significantly to the dynamics of microbial ecosystems, influencing bacterial populations and diversity. The interconnectedness of bacteriophages with bacterial communities underscores their importance in shaping microbial dynamics, with potential consequences for human health, agriculture, and environmental processes. Despite their ubiquity, the comprehensive understanding of their genetic repertoire has been relatively limited compared to that of other organisms (Clokie et al., 2011). This systematic understudy has resulted in a significant gap in our biological knowledge, particularly regarding the multifaceted roles phages play in diverse biosystems (Al- et al., 2020; Shim et al., 2021).

While historically perceived to have limited medical relevance compared to bacteria, the significance of phages in the medical domain is experiencing a resurgence. This renewed interest is closely linked to the escalating threat of antimicrobial resistance (Antimicrobial Resistance Collaborators, 2022), which has prompted a critical reassessment of alternative therapeutic strategies, notably the effectiveness of phage therapy. In the face of increasing bacterial resistance to traditional antibiotics, phages - viruses that infect and replicate within bacteria - have emerged as promising candidates for combating bacterial infections (Young and Gill, 2015; Gordillo et al., 2019). The specificity of phages in targeting particular bacterial strains, coupled with their ability to co-evolve with bacteria, presents a dynamic and potentially effective approach to counteract the challenges posed by antimicrobial resistance (Shim, 2023). This shift in perspective underscores the evolving landscape of medical research, emphasizing the importance of harnessing the unique attributes of phages in addressing the pressing global concern of antimicrobial resistance.

**FIGURE 1:**
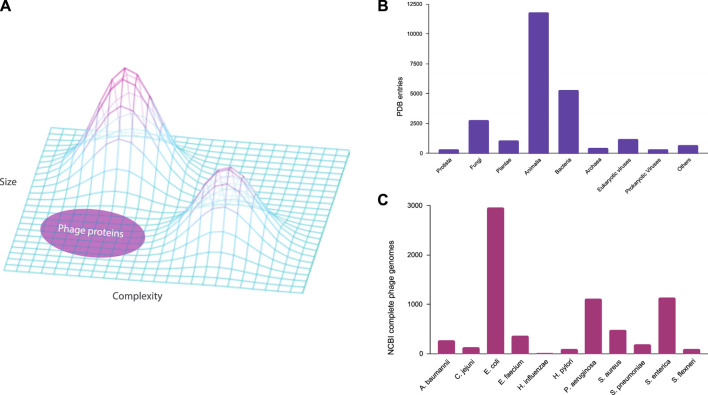
Underrepresentation of bacteriophages in the public databases. **(A)** Three-dimensional protein structure landscape represented by the variations in size and complexity. **(B)** Experimental protein structure entries by natural source organisms in the Protein Data Bank (PDB) classified at the Kingdom level. **(C)** Complete genomes of ESKAPE phages in the NCBI Virus database.

The underrepresentation of phages across various datasets not only impedes our capacity to unravel the complex dynamics of microbial interactions in nature but also introduces biases in diverse biological models. A majority of these models, reliant on current genomic data, may inadvertently incorporate a skewed perspective, thereby limiting their accuracy and applicability. This biased outcome is analogous to the recent issues in computer vision due to dataset imbalance and bias (Deviyani, 2022; Jones et al., 2024). For example, deep learning models have made significant strides in solving the long-standing protein folding problem in biology (Dill et al., 2008). However, these models rely on experimental protein structures for training and testing, which are used in combination with genomic data for multiple sequence alignment (Jumper et al., 2021). If genomic databases and protein structure repositories exhibit biases toward certain organisms, computational models derived from these datasets may fail to accurately represent the biological landscape. Bacteriophages, renowned for their rich diversity of small proteins (Shim et al., 2021), represent a facet of the protein landscape that remains relatively unexplored within the current biological context (Figure 1A). Therefore, addressing this knowledge gap becomes imperative not only for advancing our understanding of phage biology but also for refining computational models essential for numerous scientific applications.

In this study, we examine the extent of the underrepresentation of phages in the genome and protein databases, focusing on the phages that are infecting the high-priority pathogens defined by the WHO (Shim, 2023). These phages may become medically relevant as an alternative antimicrobial therapy against ESKAPE pathogens, which is an acronym for the high-priority pathogens of *Enterococcus* spp., *Staphylococcus aureus*, *Klebsiella pneumoniae*, *Acinetobacter baumannii*, *Pseudomonas aeruginosa*, and *Enterobacter* spp. (Santajit and Indrawattana, 2016). In this study, we included 11 bacterial species from the WHO priority list and studied the phages of these ESKAPE pathogens using genome and pangenome analyses. We first analyzed the extent of biased datasets against bacteriophages in the Protein Data Bank (Berman et al., 2000) (Figure 1B), before downloading all the complete genomes of the ESKAPE phages in the NCBI Virus database for the downstream analysis (Figure 1C).

From this study, we aim to provide a quantitative analysis in understanding the extent of underrepresented datasets of phages and engage the scientific community to focus more attention on phage-related data collection given the wide implications on diverse fields from phage therapy to machine learning models. As research advances, the multifaceted roles of bacteriophages in modulating bacterial behavior, participating in microbial community dynamics, and offering therapeutic solutions continue to unfold. This evolving knowledge challenges the historical notion of limited medical relevance and positions bacteriophages as integral components of the complex microbial world with far-reaching implications for diverse fields, including medicine, ecology, and biotechnology.

## Materials and methods

### Curation of ESKAPE phage datasets

We first analyzed the underrepresentation of bacteriophages in the genome and protein databases. For the protein database, we downloaded the data distribution provided by the Protein Data Bank (PDB) (Berman et al., 2000) on the entries of experimental protein structures by natural source organisms (downloaded 2024/01/24). The definition of natural source organisms for these structures is from a natural and non-modified source. To categorize each entry to a natural source organism at the kingdom level (Figure 1B), we used a combination of human expertise and a generative AI based on the large language model (ChatGPT, 2023).

Next, we curated the ESKAPE phage genome dataset by downloading the reference genomes of bacteriophages from the NCBI Virus database. Here, we define the ESKAPE phages as bacteriophages that infect ​​the WHO priority pathogens (*Helicobacter pylori*, *Campylobacter jejuni*, *Salmonella enterica*, *Streptococcus pneumoniae*, *Haemophilus influenzae*, *Shigella flexneri*) encompassing the ESKAPE (*Enterococcus* spp., *Staphylococcus aureus*, *Klebsiella pneumoniae*, *Acinetobacter baumannii*, *Pseudomonas aeruginosa*, and *Enterobacter* spp.) (Prioritization of pathogens to guide, 2019). Some pathogens, such as *Klebsiella pneumoniae* and *Neisseria gonorrhoeae*, were omitted from the list as they do not have associated bacteriophages in the NCBI virus database. All the RefSeq genomes with the specified host were downloaded for each pathogen species as whole genome sequences and protein sequences (downloaded 2022/10/18).

The genomes of each ESKAPE phage were analyzed using several biological features and statistical measures, which are important for understanding the variability in these phage datasets. The biological features included phage ID, phage type, and DNA type, and the statistical measures included GC content, AT content, GC/AT ratio, number of proteins, and sequence length (Supplementary Table S1). These measures were summarized into the most common phage type, the most common DNA type, GC content, and the number of open-reading frames (ORF) for each host ESKAPE pathogen (Table 1).

**TABLE 1:** Summary statistics of phage genomes associated with the ESKAPE pathogens.

Host pathogen	Most common phage type	Most common DNA type	GC content (mean ± s.d.)	Number of ORF (mean ± s.d.)	Genome length (mean ± s.d.)	Most common lifestyle	Probability of most common lifestyle (mean ± s.d.)
*Acinetobacter baumannii*	Obolenskvirus	dsDNA	39.12 ± 2.55	84.76 ± 83.49	59,042 ± 53,609	Lytic	0.52 ± 0.03
*Campylobacter jejuni*	Fletchervirus	dsDNA	35.07 ± 8.19	54.92 ± 75.23	82,425 ± 57,083	Temperate	0.51 ± 0.01
*Escherichia coli*	Gequatrovirus	dsDNA	43.90 ± 6.50	75.70 ± 98.48	52,765 ± 61,362	Temperate	0.56 ± 0.08
*Enterococcus faecium*	Siphoviridae	dsDNA	36.83 ± 4.41	8.84 ± 35.86	8,185 ± 29,593	Temperate	0.51 ± 0.01
*Haemophilus influenzae*	Hpunavirus	dsDNA	39.97 ± 0.05	39.50 ± 3.54	31,932 ± 599	Temperate	0.63 ± 0.04
*Helicobacter pylori*	Schmidvirus	dsDNA	37.44 ± 1.71	17.09 ± 15.73	14,224 ± 13,342	Temperate	0.52 ± 0.01
*Pseudomonas aeruginosa*	Pbunavirus	dsDNA	56.53 ± 8.00	53.45 ± 67.50	42,401 ± 50,165	Temperate	0.52 ± 0.03
*Staphylococcus aureus*	Kayvirus	dsDNA	32.67 ± 2.80	85.50 ± 78.11	63,602 ± 54,105	Temperate	0.60 ± 0.07
*Streptococcus pneumoniae*	Siphoviridae	dsDNA	39.09 ± 2.05	42.51 ± 19.23	29,383 ± 13,721	Temperate	0.53 ± 0.02
*Salmonella enterica*	Jerseyvirus	dsDNA	46.64 ± 6.12	71.06 ± 75.33	53,119 ± 53,774	Lytic	0.53 ± 0.04
*Shigella flexneri*	Tequatrovirus	dsDNA	43.14 ± 5.87	117.44 ± 88.79	78,388 ± 54,418	Lytic	0.59 ± 0.11

**FIGURE 2:**
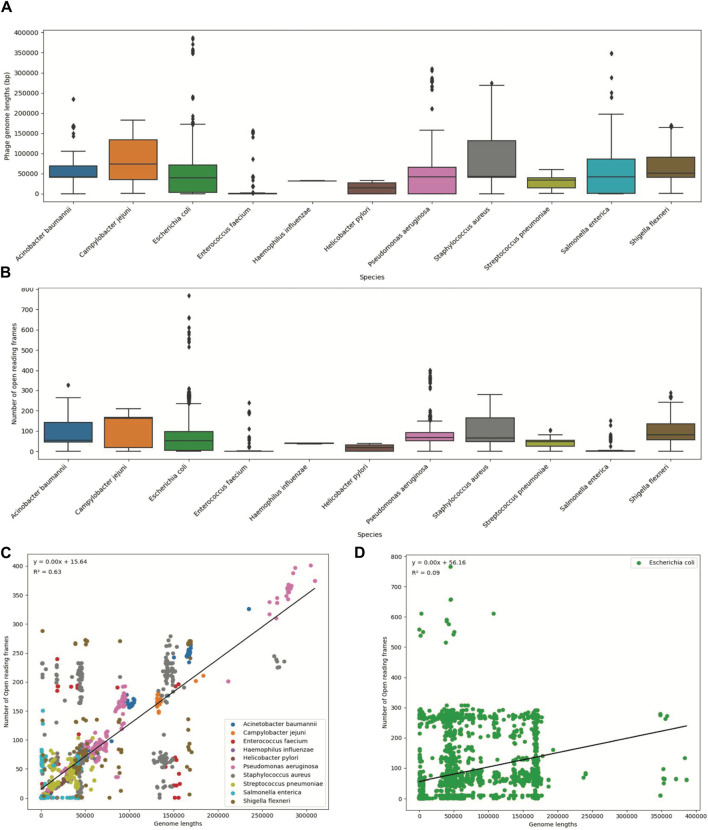
Summary statistics of the ESKAPE phage genome dataset. **(A)** Boxplot of phage genome lengths (bp) of phages by their host ESKAPE pathogen. **(B)** Boxplot of open reading frames of phages by their host ESKAPE pathogen. **(C)** Scatter plot of phage genome lengths (bp) versus open reading frames, colored by each host ESKAPE pathogen with an overlayed regression line. **(D)** Scatter plot of *E. coli* phage genome lengths (bp) versus open reading frames with an overlayed regression line.

To visualize the statistical information, we created boxplots for two variables: phage genome lengths and the number of open reading frames (Figures 2A, B). Additionally, we created scatter plots to illustrate the relationship between these two variables (Figures 2C, D). Each data point on the plot represents every ‘phage ID’, with the phage genome lengths plotted along the *x*-axis and the number of open reading frames plotted along the *y*-axis. The scatter plots included data from all 11 species, where each species was displayed in a different color. The statistics from the *E. coli* phages were plotted separately due to the high number of data points (Figure 2D). Then, we used a ‘LinearRegression’ class to fit a model that represented the linear relationship. By using this model, we calculated the regression equation and the *R* ^2^ value. The scatter plot with an overlayed regression line helped to identify whether the variables have a positive, negative, or no apparent relationship.

### Gene function analysis of ESKAPE phages

To observe the function of diverse genes in the ESKAPE phage genomes, the fasta files were processed with Biopython to extract gene information such as gene ID, virus ID, gene name, virus name, virus genus, and family. We extracted the gene keywords such as ‘DNA’ or ‘polymerase’ from the gene names. A vast majority of the gene names contain the keyword ‘hypothetical’. A hypothetical gene is a predicted gene that is likely to be expressed in organisms, but which has never been characterized for biochemical function (Galperin, 2001). Therefore, keywords related to hypothetical genes were excluded from the subsequent processing.

To visualize these gene keywords and names, we generated a keyword heatmap and a horizontal bar chart for each ESKAPE gene genome, respectively. The genes with the keyword ‘putative’ also have unknown functions like hypothetical genes, but they have similar properties to already existing genes (Alexandre et al., 1988). Since they are assumed to be functional genes, this keyword was kept for further study. The visualization of these genes was done in three sets; the first set includes only functional genes excluding hypothetical and putative genes (Supplementary Figure S1), the second set includes only putative genes (Supplementary Figure S2), and the third set includes all functional and putative genes (Supplementary Figure S3).

**FIGURE 3:**
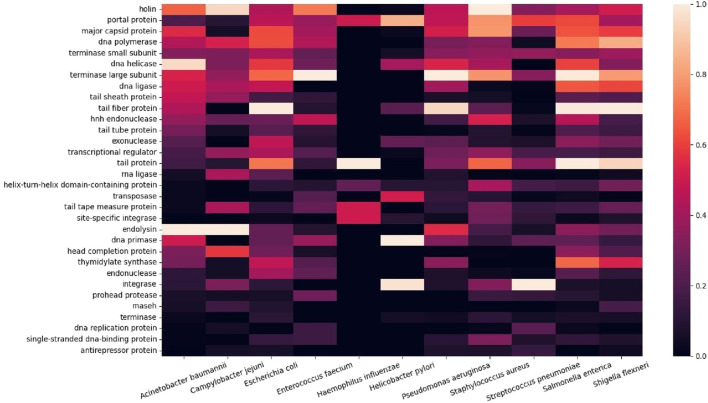
Heatmap of co-occurring functional genes from the ESKAPE phage genomes.

Subsequently, we summarized the gene functions into a heatmap to compare the relative magnitudes of genes co-occurring in the ESKAPE phage genomes (Figure 3). Gene keywords from each ESKAPE gene genome were downloaded as CSV files, which were processed into ‘word’ and ‘value’ columns. To calculate the frequency of common words within 22 CSV files (11 CSV files were downloaded from each set: all genes and only functional genes), we used the ‘Counter’ object. Each word was treated as a unique key and its occurrences are computed individually, for example, ‘holin’ or ‘putative holin’. We created a list of regular expression patterns by selecting the 32 words with the highest frequencies from the extracted unique keys. Each target word in the list was processed using '\b{}\b' to indicate a word boundary in the regular expression pattern. These patterns were then used to search for words in the previous CSV files that fully matched the pattern. Finally, we merged all the data into a single data frame and generated the heatmap, which provided us with a visual pattern to easily grasp the relationships between the data.

### Genome visualization and phage lifestyle prediction

We used a genome viewer to visualize the genomic architecture of each phage genome. In this process, the untranslated reference sequence genome fasta file and translated reference sequence protein fasta file were separated into every single IDs of phages. The translated reference sequence protein fasta file contains the information about IDs of phage and locations where the protein of each phage is translated. To simplify reference sequence protein fasta files, phage IDs were extracted from the untranslated reference sequence genome fasta files through Python. Using the phage IDs in the list, new files containing all translation sites per phage ID were created. With the DNA feature viewer algorithm, linear graphs were made with whole genome data while omitting hypothetical proteins (Supplementary Figure S4).

PHACTS is a computational approach that classifies the lifestyle of bacteriophages (McNair et al., 2012). The lifestyle of a phage can be classified as virulent (lytic) or temperate (lysogenic). For the virulent and temperate types, a phage genome is annotated as either ‘Lytic’ or ‘Lysogenic’, respectively, with a computed probability. PHACTS predicts the lifestyle of a bacteriophage based on the genome content through two training sets and the Random Forest method (Ho, 2024a; Ho, 2024b). One of the training sets of the query protein sequences was selected from the newly edited fasta files. The other training set is based on the known phage sequence data from PHANTOME (Batstone et al., 2022), providing a complete phage genome with two training sets. The Random Forest algorithm of PHACTS classifies the lifestyle of phages by creating multiple decision trees. Then, the known phage sequence data is loaded into decision trees through the bootstrapping method, which randomly picks data by resampling. Randomly selected query protein sequences from the newly edited fasta files are matched with the known phage sequence data to complete the decision trees. Finally, the tree with the highest computed probability is chosen as the final prediction model. To keep the accuracy and stable runtime, replicate iterations are executed 10 times. Among 10 iterative replicates, the sole consensus result values are determined as the confident data (McNair et al., 2012).

### Pangenome analysis of ESKAPE phage genomes

**FIGURE 4:**
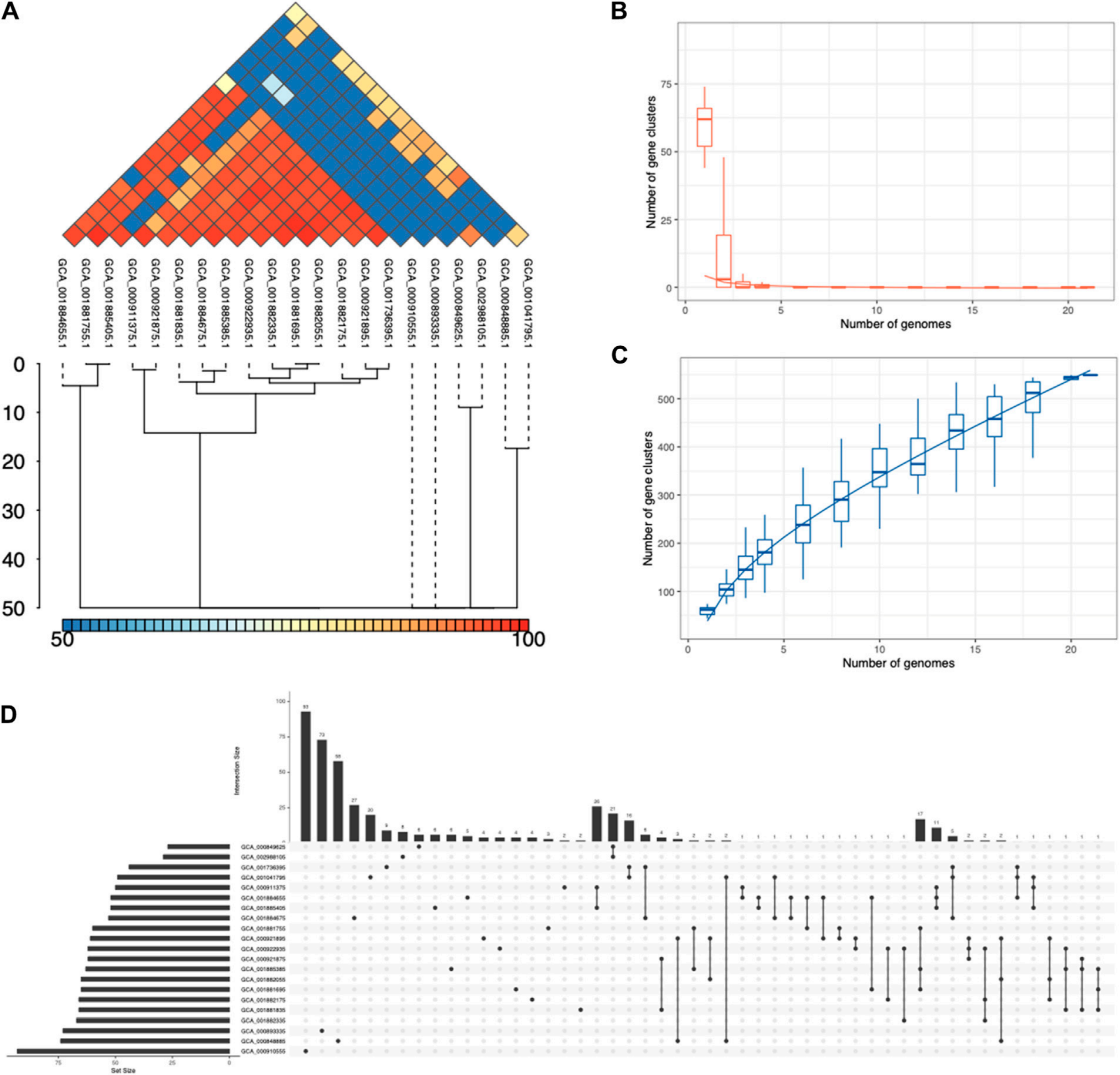
Pangenome analysis of the ESKAPE pathogens. **(A)** Average Nucleotide Identity (ANI) in *S. pneumoniae* phages. **(B)** Core genome clusters in *S. pneumoniae* phages. **(C)** Pangenome clusters in *S. pneumoniae* phages. **(D)** Set sizes and intersection sizes in *S. pneumoniae* phages.

We used IPGA (v1.09) to analyze, compare, and visualize the pangenome of ESKAPE phages (Liu et al., 2022). This web tool features a scoring system that evaluates the reliability of profiles generated by different pangenome methods. We ran several pangenome packages with the phages of *Salmonella enterica* as an initial trial. Using IPGA, we compared the pangenome profiles created by different methods and found that PEPPAN (Zhou et al., 2020) was the only pangenome software that performed well with the ESKAPE phage genomes as input data. Thus, all the other phage genomes of the ESKAPE pathogens were analyzed using PEPPAN as the pangenome analysis option (Figure 4; Supplementary Figure S5). As pangenome analysis requires four or more individual genomes, the phages of *H. influenzae* with only two individual genomes were excluded from the pangenome analysis. In addition, IPGA also implements several downstream comparative analysis modules and genome analysis modules, including Average Nucleotide Identity (ANI) that measure nucleotide-level genomic similarity between the coding regions of genomes (Konstantinidis and Tiedje, 2005).

MMseqs2 is a deep learning-based software to search and cluster huge sequence sets, with a highly efficient clustering module to group similar sequences into clusters (Steinegger and Söding, 2017). For clustering whole-genome sequences of these ESKAPE phages, we used a clustering module of MMseqs2 that is highly efficient at grouping similar sequences into clusters. It employs an iterative clustering approach, progressively merging sequences into clusters while optimizing a predefined objective function to achieve accurate and scalable clustering results. We created a database containing the genomes of phages against each ESKAPE pathogen and clustered these genomes with a minimum sequence identity of 70%.

### Clustering of phage protein sequences by similarity

For clustering protein sequences, a clustering in linear time called the Linclust algorithm in MMseqs2 was used for fast clustering, which reduces the time complexity $O\left(NK\right)$ to $O\left(N\right)$ , where K is the parameter that indicates the final number of clusters. The Linclust algorithm operates by generating a table that consists of the k-mer, the sequence identifier, and the sequence position, and sorting the table by k-mer to identify groups of sequencing sharing the same k-mer in quasi-linear time. Then, the longest sequence is selected as the center sequence among the sequences that share the same k-mer. The groups are merged around the center sequence and the sequence groups are compared by the global Hamming distance and gapped local sequence alignment. Finally, the representative sequence and aligned sequences are determined through the incremental greedy algorithm.

We used two settings of sequence identity thresholds (50% and 90%, respectively) for clustering as shown below. For the sequence identity of 50%, the mode was set as 1 which enables the alignment to cover at least 50% between query and target.

mmseqs easy-cluster examples/DB.fasta clusterRes tmp --min-seq-id 0.5 -c 0.5 --cov-mode 1

For the sequence identity of 90%, the mode was set as 0 which enables the alignment to cover at least 80% between query and target.

mmseqs easy-cluster examples/DB.fasta clusterRes tmp --min-seq-id 0.9 -c 0.8 --cov-mode 0.

We compared the results from these two sets with the different sequence identity thresholds. We determined that the sequence identity threshold of 90% was too stringent, thus the following structure analysis was conducted with the set with the sequence identity threshold of 50%. The representative sequences were extracted from the resulting MMseqs2 file for AlphaFold structure prediction.

### AlphaFold-predicted structures of representative inhibitor phage proteins

**FIGURE 5:**
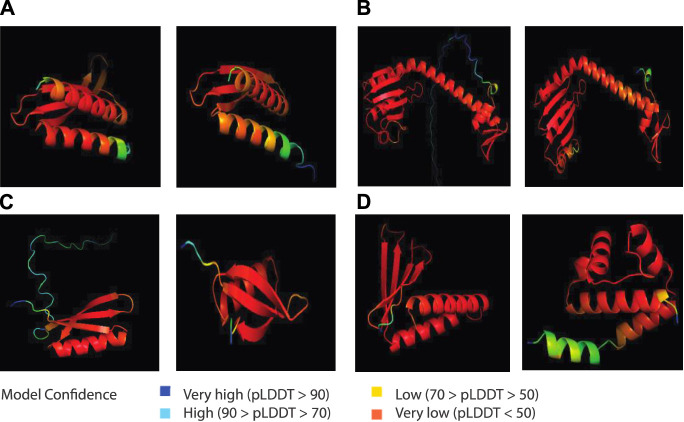
AlphaFold-predicted inhibitor proteins from lytic phages of the ESKAPE pathogens. **(A)** Inhibitors of host energy from *S. enterica* phages. **(B)** Inhibitors of host transcription from *E. coli* phages and *S. enterica* phages. **(C)** Inhibitors of host translation from *S. enterica* phages. **(D)** Inhibitor of host toxin/antitoxin system from *S. enterica* and anti-sigma factor from *E. coli* phages. The pLDDT score represents the model’s estimate of its performance on the Local Distance Difference Test.

AlphaFold is a deep learning-based program that could predict three-dimensional structures of proteins (Jumper et al., 2021). We used AlphaFold to gain insight into the structure of representative inhibitor phage proteins (Figure 5; Supplementary Figure S6). We performed AlphaFold using Google Colab and generated protein structure predictions from the representative protein sequences. To identify only the inhibitor proteins of lytic phages, we selectively screened lytic phages of the ESKAPE pathogens (Supplementary Table S2) and identified the ‘Phage ID’ of the lytic phages that corresponded to the ‘Protein ID’ of the inhibitor phage proteins (Supplementary Table S3).

Subsequently, the PDB files of the predicted protein structures were downloaded, and we observed the three-dimensional representation of protein structures with the Pymol, including alpha helices, beta sheets, and tertiary structures. In addition, we analyzed the temperature factor column in the PDB file describing the per-residue Local Distance Difference Test (pLDDT) of each residue (Jumper et al., 2021). The pLDDT corresponds to the model’s estimate of its score on the local Distance Difference Test (lDDT-Cα), which is a measure of local accuracy. These scores are represented on a spectrum, with higher certainty depicted in red and lower certainty shown in blue.

## Results

### Bacteriophages are highly underrepresented in the databases

In the Protein Data Bank (PDB), we discovered that the experimental structures of bacteriophage proteins are highly underrepresented when classified at the kingdom level (Figure 1B). The bar chart of the PDB entries by natural source organism shows that the kingdom of Animalia is highly represented, particularly biological model organisms such as *Homo sapiens* (3,364 entries) and *Mus musculus* (1,265 entries). Furthermore, the kingdoms that contain pathogens against humans such as Bacteria and Eukaryotic viruses are well represented in the PDB database with entries of 5,262 and 1,165, respectively. The least represented kingdoms are Protista, Archaea, and Prokaryotic viruses (i.e., bacteriophages) with entries of 294, 415, and 309, respectively. For the ESKAPE phages, we found only few to no entries for *Acinetobacter baumannii* (0), *Campylobacter jejuni* (0), *Escherichia coli* (73), *Enterococcus faecium* (0), *Haemophilus influenzae* (0), *Helicobacter pylori* (4), *Pseudomonas aeruginosa* (10), *Salmonella enterica* (12), *Shigella flexneri* (4), *Staphylococcus aureus* (8), *Streptococcus pneumoniae* (3). Even considering that the protein contents of these organisms differ vastly, as the full human genome contains 24,000 proteins while a typical phage genome contains fewer than 100 proteins (Table 1), this underrepresentation of phage proteins in the PDB is still striking. For example, eukaryotic viruses with a similar number of proteins have 1,165 entries as compared to 309 entries of prokaryotic viruses in the PDB.

For the genome database, this study reveals a paucity of complete phage genomes associated with the WHO priority list pathogens in the NCBI Virus database (Figure 1C). For example, *H. influenzae*, a Gram-negative bacterium causing pneumonia, meningitis, or bloodstream infections (Fleischmann et al., 1995), has only two known complete phage genomes. Furthermore, these phages exhibit temperate lifestyles (Table 2), limiting their suitability for phage therapy applications. Despite the evident advantages of phage therapy, several challenges persist, especially in the realm of fundamental research. A notable challenge is the inadequate comprehension of the diversity of lytic bacteriophages within their natural habitats, such as human microbiomes. This scarcity of diverse lyric phage genomes poses a substantial impediment to the development of effective phage therapy treatments tailored to specific bacterial infections.

**TABLE 2:** Summary statistics of lytic phage genomes associated with the ESKAPE pathogens.

Host pathogen	Most common phage type	Most common DNA type	GC content (mean ± s.d.)	Number of ORF (mean ± s.d.)	Genome length (mean ± s.d.)	Most common lifestyle	Probability of most common lifestyle (mean ± s.d.)
*Acinetobacter baumannii*	Friunavirus	dsDNA	38.91 ± 2.69	112.40 ± 101.31	78,735 ± 64,666	Lytic	0.53 ± 0.04
*Campylobacter jejuni*	Fletchervirus	dsDNA	37.01 ± 8.44	48.10 ± 78.53	88,282 ± 58,035	Lytic	0.51 ± 0.01
*Escherichia coli*	Gequatrovirus	dsDNA	42.28 ± 6.25	73.97 ± 97.41	61,027 ± 68,482	Lytic	0.58 ± 0.10
*Enterococcus faecium*	Siphoviridae	dsDNA	37.16 ± 4.07	7.71 ± 33.24	9,579 ± 33,455	Lytic	0.51 ± 0.01
*Haemophilus influenzae*	N/A	N/A	N/A	N/A	N/A	N/A	N/A
*Helicobacter pylori*	Schmidvirus	dsDNA	37.77 ± 1.65	6.90 ± 12.40	5,579 ± 10,797	Lytic	0.51 ± 0.01
*Pseudomonas aeruginosa*	Pbunavirus	dsDNA	56.26 ± 8.16	35.36 ± 46.91	31,593 ± 36,682	Lytic	0.52 ± 0.03
*Staphylococcus aureus*	Kayvirus	dsDNA	30.83 ± 2.66	107.89 ± 88.85	94,240 ± 61,839	Lytic	0.57 ± 0.06
*Streptococcus pneumoniae*	Cepunavirus	dsDNA	37.76 ± 4.25	29.60 ± 28.04	17,318 ± 15,731	Lytic	0.52 ± 0.04
*Salmonella enterica*	Kuttervirus	dsDNA	45.48 ± 6.37	12.52 ± 27.69	66,567 ± 61,489	Lytic	0.53 ± 0.04
*Shigella flexneri*	Tequatrovirus	dsDNA	41.46 ± 5.76	133.92 ± 96.42	96,430 ± 57,962	Lytic	0.62 ± 0.12

Boxplots provided us with a concise overview of statistics (distribution, median, quartiles, and presence of outliers), allowing for easy comparison and interpretation (Figure 2). The boxplots of the phage genome lengths by the host show that most phages have genome lengths below 50 kbp (Figure 2A). Notably, the phages of *E. coli*, *P. aeruginosa*, and *S. enterica* have high variations in the genome length, with some phages reaching above 200 kbp in length (Michniewski et al., 2021). The boxplots of the number of ORFs by the host show that most phages have protein lengths below 100 bp (Figure 2B). Notably, the phages of *E. coli* and *P. aeruginosa* have high variations in the protein length, with some proteins reaching above 400 bp in length. This result shows that most ESKAPE phages have small genome lengths packed with small proteins (Fremin et al., 2022; Silpe et al., 2023). The scatter plots with an overlayed regression line show that the relationship between the genome length and the number of ORFs is positive, but this relationship only has a coefficient of determination, or *R* ^2^, value of 0.63 for the ESKAPE phages, except that of a much lower value of 0.09 for the *E. coli* phages as shown in Figures 2C, D, respectively. This *R* ^2^ value measures the goodness of fit of this regression model, and it shows that only 63% of the variance in the number of ORFs can be explained by the genome length variables in the ESKAPE phages, except in the *E. coli* phages with a much lower value at 9%. The variation of the number of ORFs versus the genome length appears to be much higher in the *E. coli* phages, with only many small phages having a larger number of proteins than expected by the regression model, and *vice versa* (Figure 2D).

### Some ESKAPE phages have genes with antimicrobial activities

As the first exploratory analysis, we created heatmaps visualizing the top keywords in the annotations of the ESKAPE phage genomes. We searched for keywords containing ‘inhibit’ or ‘anti’ that are potentially related to the antimicrobial activities of phages against their hosts. The ESKAPE phage with the most antimicrobial keywords such as ‘inhibit’ or ‘anti’ within the functionally validated genes was associated with *E. coli* (Supplementary Figure S1). Even after adjusting for the large dataset of available genomes, the *E. coli* phages have 10 or 20 times more genes with the antimicrobial keywords as compared to the *P. aeruginosa* phages with half the available genomes. The phages of *S. enterica* contain the next most abundant genes associated with antimicrobial activities. Notably, the phages of *H. pylori* and *C. jeuni* have almost no functional genes with antimicrobial activities, which may be explained by the lack of available phage genomes in the NCBI Virus database (Figure 1). However, the phage genomes of *C. jejuni* contain a small number of putative antimicrobial genes, including ‘anti-holin’, in the putative gene list (Supplementary Figure S2).

This finding remains the same when the putative genes were included in the analysis (Supplementary Figure S2). The putative genes containing the keywords ‘inhibit’ or ‘anti’ show the potential for novel antimicrobial activities from the ESKAPE phages. For example, the ‘anti-proliferative’ gene only appears in the list of putative genes, and, interestingly, this gene may be involved in the regulation of cell growth and development (Kalie et al., 2007; Zhang et al., 2023). Subsequently, we analyzed the most frequent co-occurrence of functional proteins in the ESKAPE phage genomes (Figure 3). The heatmap shows the top 30 functional proteins that are shared between the ESKAPE phage genomes in this study. This heatmap shows that most annotations are related either to the replication function of bacteriophages, such as polymerase and helicase, or to the assembly function of bacteriophages, such as capsid and tail.

Finally, we analyzed the gene content of these phages further examining individual genes through bar graphs (Supplementary Figure S3). The bar charts show the 30 most frequent genes in each ESKAPE phage. These bar charts reveal some genes of interest may have inhibitory functions. Several ESKAPE phages have several antimicrobial genes that may disrupt the vital functions of host bacteria. For example, *A. baumannii*, *E. coli*, *S. enterica*, and *S. flexneri* have anti-sigma factors that bind to sigma factors and inhibit transcriptional activity in regulating prokaryotic gene expression (Paget, 2015). Moreover, *E. coli*, *S. enterica*, and *S. flexneri* have host polymerase inhibitors that may disrupt the transcriptional activities of host bacteria (Pilotto et al., 2021). These genes are likely to be antibacterial as the disruption of transcription leads to cell cycle arrest (Merrikh et al., 2023). These phages also possess other genes that are involved in counter-defensive activities against the host defense mechanisms, such as anti-restriction genes (Spoerel et al., 1979), host protease inhibitors (Meyn et al., 1977), anti-repressor (Horiuchi et al., 1974), and anti-crispr genes (Bondy-Denomy et al., 2012; Park et al., 2022a; Park et al., 2022b; Shim, 2022).

#### Databases have a limited number of lytic ESKAPE phages

We visualized the genomic architecture of the ESKAPE phages to explore the gene content. The genome maps of these ESKAPE phages revealed that many of these phages have similar gene content, and many of these phages are nearly identical. Remarkably, these phage genomes also share taxonomic relationships, resulting in even fewer unique genomes for potential therapeutic use. For instance, our analysis indicates two phage genomes infecting *H. influenzae* are nearly identical in gene content (Supplementary Figure S4).

Given the lack of antimicrobial genes in the ESKAPE phage genomes, we explored the lifestyle of these phages (Table 2; Supplementary Table S2). Identifying the lifestyle of a phage traditionally relies on labor-intensive and costly culturing techniques. However, these methods are not only time-consuming but also impractical for phage genomes derived from environmental sequencing. We used a computational approach to predict the lifestyle of the ESKAPE phages (McNair et al., 2012). Phages demonstrate two main lifestyles: virulent (lytic) and temperate (lysogenic). This computational approach leverages the gene content inherent to phage genomes to predict the lifestyle of a phage.

According to the computational prediction, there is only a limited number of lytic phages against all the ESKAPE pathogens (Supplementary Table S2). For example, the phages of *C. jejuni*, *E. faecium,* and *H. pylori* only have one lytic phage in the database. Other ESKAPE pathogens such as *A. baumannii* and *S. pneumoniae* also have four and two lytic phages, respectively. More importantly, *H. influenzae* has no lytic phages that are predicted by the computational method. The medical importance of bacteriophages lies in their potential applications in phage therapy, a field that explores the use of bacteriophages to combat bacterial infections. Bacteriophages exhibit specificity in targeting bacterial strains, making them attractive candidates for precision medicine in treating bacterial infections. Their ability to infect and lyse bacteria provides a natural and tailored approach to controlling pathogenic bacteria, including antibiotic-resistant strains. The biological features of the lytic phages were summarized into the most common phage type, the most common DNA type, GC content, and the number of open-reading frames (ORF) for each host ESKAPE pathogen (Table 2). The curated dataset of bacteriophages with lytic lifestyles that are potential candidates for phage therapy is shared for each class of ESKAPE pathogen (Supplementary Table S2).

### Pangenome analysis shows the ESKAPE phages share many core genes

For the ESKAPE phages that share the same host, we used the clustering method to automatically cluster their genomes by similarity at the nucleotide level. When we clustered these phages with the minimum sequence identity of 70%, it revealed that the number of individual phages can be reduced into a small number of clusters. For example, 263 individual phages infecting *A. baumannii* can be reduced into 3 clusters with the clustering method (Figure 4A). This shows that the NCBI virus database only contains 3 unique phages against the high-priority pathogen of *A. baumannii*. Similarly, the genome maps of phages infecting *A. baumannii* can also be divided into a few categories based on visualization (Supplementary Figure S4). Notably, many phages share the same gene content and even the same gene arrangement. However, the public databases do not compute the similarity between these phages as there is no consensus on how to classify these phages into different ‘strains’.

The objective of pangenome analysis is to assess the diversity of all genes and genomic structures across genomes within a particular clade. The primary and pivotal step in this analysis involves clustering orthologous genes. These gene clusters are subsequently categorized into three groups based on their presence in the specified sets of genomes: core genes, accessory genes, and unique genes. Multiple packages or web services have been developed for pangenome analyses of eukaryotic and prokaryotic genomes (Li et al., 2003; Fouts et al., 2012; Santos et al., 2013; Page et al., 2015; Chen et al., 2018; Ding et al., 2018; Emms and Kelly, 2019; DeSalle et al., 2020; Gautreau et al., 2020; Tonkin-Hill et al., 2020; Zhou et al., 2020; Galperin et al., 2021), but we found no method that is specifically designed for phage genomes. We chose an integrated prokaryotes genome and pangenome analysis web service called IPGA that allows phage genomes as input for pangenome analysis, downstream analysis, and visualization of the target genomes (Liu et al., 2022).

The first step of IPGA was a quality control module that removes all low-quality genomes and performs a taxonomic assignment for each genome. IPGA then predicted genes of all filtered genomes and used them as the input of the pangenome analysis module (Figure 4; Supplementary Figure S5). Not all phage genomes of the ESKAPE pathogen had pangenome analysis results, such as *H. influenzae* and *E. faecium*, due to the lack of individual genomes. The ESKAPE phages of *E. coli* and *S. pneumoniae* failed to give any meaningful results due to the large input dataset. The pangenome results show that the ESKAPE phage genomes have a high number of core genes that are shared as the intersection between the phages infecting the same host. For example, the core genome clusters in *S. pneumoniae* phages decrease rapidly (Figure 4B) while the pangenome clusters in *S. pneumoniae* phages increase slowly (Figure 4C). This relative difference in the incline and decline rates indicates that more core genes are shared between the phage genomes of this pathogen than the accessory or unique genes. The set sizes and intersection sizes also reflect the size of the core genome versus the size of the pangenome in the *S. pneumoniae* phages (Figure 4D). This trend is observed in the pangenome analysis of the other ESKAPE phages, including *C. jejuni*, *H. pylori*, and *S. flexneri* (Supplementary Figure S5).

We also conducted the downstream comparative genomic analysis modules on the filtered genomes and gene clusters, including the phylogenetic analysis module, core gene allele analysis, and average nucleotide identity (ANI) calculation module. Average Nucleotide Identity (ANI) is a measure of nucleotide-level genomic similarity between the coding regions of two genomes. The ANI statistics show that many phage genomes share high genomic similarity at the nucleotide level. For example, the ANI analysis of *S. pneumoniae* phages shows that there are 6 clusters of phage genomes with genomic similarity below 70% at the nucleotide level (Figure 4A).

#### Protein structure analyses show the underexplored structure landscape

After the pangenome analysis of the ESKAPE phage genomes, we clustered the functional and putative protein sequences with antimicrobial activities. Subsequently, we predicted the three-dimensional protein structure of a representative protein from each cluster. When these representative proteins were visualized, we found that these proteins share several secondary and tertiary structural components despite being in different clusters in terms of genetic sequence (Supplementary Figure S6). For example, the inhibitors of host energy from *S. enterica* phages share similar tertiary structures (Figure 5A). Furthermore, the inhibitors of host transcription from *E. coli* phages and *S. enterica* phages also share similar tertiary structures (Figure 5B). Also, the inhibitors of host translation from *S. enterica* phages follow the same trend (Figure 5C). Interestingly, small proteins such as the inhibitors of the host toxin/antitoxin system from *S. enterica* and the anti-sigma factors from *E. coli* phages have unique structures (Figure 5D). This protein structure analysis of the representative proteins with potential antimicrobial activities reveals the underexplored surface of the protein structure landscape, which are small proteins with diverse structural components (Figure 1A).

AlphaFold generates a per-residue model confidence score known as pLDDT, ranging from 0 to 100, where regions scoring below 50 pLDDT may lack structural integrity when considered in isolation. Notably, the predicted structures of all the proteins exhibit consistently low confidence levels, predominantly below the threshold of 50 pLDDT across various regions (Figure 5). This pattern suggests limitations in AlphaFold’s ability to accurately predict the structures of phage proteins. A probable contributing factor could be the relatively small number of phage proteins included in the training dataset of this computational model. This observation underscores the importance of expanding the diversity of proteins represented in training datasets to improve the accuracy and applicability of structure prediction algorithms like AlphaFold. Particularly, there is a notable gap in the exploration of antimicrobial proteins derived from lytic phages, which are increasingly recognized for their potential role in phage therapy (Shim, 2023). Despite recent findings highlighting their significance, these proteins remain understudied, as evidenced by the limited confidence in their predicted structures.

## Discussion

The underrepresentation of bacteriophages in biological databases despite being the most abundant biological entities in the biosphere raises concerns that a significant portion of this vital biosphere remains unexplored. This study seeks to quantify the extent of the underrepresentation of phage data within public databases and to discuss the implications of such biased datasets. Specifically, it aims to assess the impact on medically relevant fields such as phage therapy, as well as on data-driven computational models like deep learning-based protein structure prediction programs. In this study, we revealed the extent of underrepresentation of bacteriophages in the Reference Sequence (RefSeq) of the NCBI Virus. This database provides a comprehensive and non-redundant set of sequences and a stable reference for genome annotation, forming a foundation for medical and functional studies. We examined the phage genomes infecting 11 bacterial species categorized under the ESKAPE acronym, designated as the top priority pathogens by the WHO for new drug development due to widespread multidrug resistance (Shim, 2023). The gene content analysis shows that several ESKAPE pathogens, such as *C. jejuni*, *E. faecium*, and *H. influenzae*, have only a few phages that are likely to have antimicrobial activities. Notably, *H. pylori* has no unique phages with functionally annotated genes encoding for antimicrobial activities (Supplementary Figure S1). More importantly, these bacteria have no putative antimicrobial genes (Supplementary Figure S2).

Next, we conducted the lifestyle analysis of these ESKAPE phages to confirm that there is only a handful of lytic phages for most pathogens (Table 2). The lifestyle of bacteriophages holds profound implications across various fields, including phage therapy, genomics, and microbiology. In the lytic lifecycle, the phage infects a bacterial host cell, hijacks its machinery to replicate its own genetic material and produce progeny phages, and ultimately causes the host cell to burst, releasing the newly formed phages to infect neighboring cells. This process results in the immediate destruction of the host cell (Shim et al., 2021). On the other hand, in the lysogenic lifecycle, the phage inserts its genetic material into the host cell’s genome, becoming a prophage. The prophage is replicated along with the host cell’s DNA during cell division, remaining latent within the host cell without causing immediate harm. Under certain conditions, such as exposure to stressors, the prophage may become activated, entering the lytic cycle and causing the host cell to lyse (Knowles et al., 2016). The lack of lytic phages in the genomic repertoire reduces the number of treatment options in phage therapy (Shim, 2023). Phage therapy, leveraging the natural predation of bacteriophages on bacteria, is gaining traction as a viable alternative or complementary strategy to traditional antibiotics (Young and Gill, 2015; Gordillo et al., 2019). As antibiotics struggle to maintain efficacy against evolving bacterial defenses, bacteriophages offer a tailored and evolving solution, capable of adapting to bacterial mutations (Shim, 2023).

Given the lack of phages with antimicrobial activities, we used the pangenome analysis to reveal how many of the ESKAPE phages are unique in terms of nucleotide identity and gene content. From whole-genome clustering and average nucleotide identity computation, we found that the ESKAPE phages of the same host share many core genes. Additionally, the ESKAPE phages form a small number of clusters in the whole-genome phylogenetic analysis. The average nucleotide identity analysis also highlights a deficiency in unique phage genomes within the reference sequence database, which ideally should offer a comprehensive and non-redundant collection of sequences. To enhance the classification of phage genomes, we propose the adoption of clustering methods that prioritize gene content rather than gene arrangement, considering the rapid evolutionary mechanism of phages.

Last, we used the deep learning-based structure program to predict the three-dimensional structures of the representative proteins with potential antimicrobial activities in the ESKAPE phages. After clustering with the multiple sequence alignment method, we selected representative sequences for each cluster (Supplementary Figure S6). From the visualization, we found that the ESKAPE phages only possess a few antimicrobial proteins with unique structures (Figure 5). Notably, the unique structures of these antimicrobial proteins are mostly small proteins such as anti-sigma proteins or toxin/antitoxin proteins. The protein structure landscape is not complete without exploring the small proteins from phages (Figure 1A). These small proteins derived from phages are increasingly recognized for their remarkable diversity (Jumper et al., 2021; Park et al., 2022b).

The sequencing of bacteriophages presents distinct challenges compared to other microbial entities, such as bacteria and archaea. Bacteriophages exhibit an extraordinary degree of genetic diversity. Unlike bacteria and archaea, for which reference genomes are relatively abundant, the lack of comprehensive reference databases for bacteriophages complicates the alignment and assembly processes during sequencing (Shim, 2019a). Bacteriophages exhibit rapid rates of evolution, leading to genomic changes that occur over short periods (Shim, 2019b). This evolutionary dynamism can complicate the assembly and analysis of phage genomes, particularly when attempting to capture the full spectrum of genetic variation. Environmental samples often contain a multitude of different bacteriophages, and some phages can even infect the same bacterial host. This phenomenon, known as mixed infections, introduces complexities in the interpretation of sequencing data, making it challenging to distinguish individual phage genomes in a mixture (Mathew et al., 2019). Other challenges arise from a combination of factors, including limitations in phage-specific extraction protocols and computational tools. Addressing these challenges in bacteriophage sequencing requires the development of specialized protocols, bioinformatics tools, and reference databases tailored to the unique characteristics of these viral entities.

Lastly, we emphasize the implications of biased datasets on data-driven computational models, particularly deep learning-based tools that have been trained on the currently available datasets for decision-making and generative processes (Jumper et al., 2021; Park et al., 2022b). The challenge of underrepresented datasets poses a substantial concern, particularly in the context of machine learning models. This issue becomes particularly pronounced when the training data used to train these models is biased, leading to skewed and potentially inaccurate results during the model’s predictive or classification tasks. In machine learning, the efficacy and reliability of a model are highly contingent on the quality and representativeness of the data it is trained on. When certain groups or categories within the dataset are underrepresented, the model may not adequately learn the patterns and characteristics associated with those groups (Deviyani, 2022; Jones et al., 2024). This lack of representation can lead to a biased understanding of the data, resulting in a model that is less capable of making accurate predictions or classifications for the underrepresented groups. The consequences of biased training data are far-reaching and can manifest in various ways. For instance, in predictive modeling of protein structures, the model may struggle to generalize well to instances that belong to underrepresented classes, leading to poor performance for phage proteins.

Bacteriophages have undergone a paradigm shift in recent years. Initially perceived as having limited medical relevance compared to bacteria, bacteriophages are now recognized as essential players in various medical and therapeutic contexts. This shift in perspective is primarily attributed to a deeper understanding of the intricate relationships between bacteriophages and their bacterial hosts. Recent advances in sequencing technologies and methodologies are continually improving our ability to overcome these obstacles and unravel the genetic intricacies of bacteriophages (Park et al., 2023). In our future research endeavors, we are dedicated to tackling the issue of underrepresentation of phage-related data through comprehensive genome sampling initiatives, leveraging the latest advancements in long-read sequencing technology. By harnessing the capabilities of long-read sequencing technologies, we aim to overcome the inherent limitations of traditional short-read sequencing methods, which often fail to capture the full complexity and diversity of phage genomes.

## Data Availability

Publicly available datasets were analyzed in this study. This data can be found here: https://github.com/hshimlab.
