# Supplementary material for: A pangenome analysis of ESKAPE bacteriophages: the underrepresentation may impact machine learning models
Source: Front Mol Biosci. 2024 Jun 21;11:1395450. doi: 10.3389/fmolb.2024.1395450 (PMC11224154; doi:10.3389/fmolb.2024.1395450)
Supplement: Supplementary file 1 [file DataSheet1.pdf]

Supplementary Information: Tables and Figures

A pangenome analysis of ESKAPE bacteriophages: the underrepresentation may impact machine learning models

Jeesu Lee[1], Branden Hunter[2], Hyunjin Shim[1,2]

Author Information

Affiliations

[1]Center for Biosystems and Biotech Data Science, Ghent University Global Campus, Incheon 21985, South Korea
[2]Department of Biology, California State University, Fresno, 5241 N Maple Ave, Fresno, CA 93740, USA

*Corresponding author: Hyunjin Shim (shim@csufresno.edu)

Figure S1. The keyword heatmap is generated from the keywords in the functional genes associated with each ESKAPE phage species. The color of each word is representative of the frequency of each keyword in the gene name.

	A. baumannii	*C. jejuni*	*E. coli*	*E. faecium*	*H. influenzae*	*H. pylori*	*P. aeruginosa*	*S. aureus*	*S. pneumoniae*	*S. enterica*	*S. flexneri*
**inhibit**	112		1724				55	63		189	91
**anti-sigma**	43		372	1						25	29
**anti-restriction**			325				5			23	4
**anti-repressor**	16	3	211	2			30		28	27	5
**anti-termination**			305				5			94	16
**anti-terminator**			33							2	2
**anti-prophage**			4								2
**anti-holin**	10		62				2			10	2
**anti-recbcd**			6							18	1
**anti-toxin**			86		6		13		5	3	1
**anti-receptor**									5	7	
**anti-freeze**			3							1	
**anti-porter**										1	
**anti-crispr**							20				
**anti-bacterial**			15	2							
**anti-immunity**			13								
**anti-dote**			4								
**anti-adapter**			4								
**anti-oxidant**		1									

Figure S2. The keyword heatmap is generated from the keywords in the putative genes associated with each ESKAPE phage species. The color of each word is representative of the frequency of each keyword in the gene name.

	A. baumannii	*C. jejuni*	*E. coli*	*E. faecium*	*H. influenzae*	*H. pylori*	*P. aeruginosa*	*S. aureus*	*S. pneumoniae*	*S. enterica*	*S. flexneri*
**inhibit**	73	1	141				33			16	4
**anti**			3					1			
**anti-sigma**	13		191				1	2			4
**anti-restriction**	1		21				1				2
**anti-repressor**	2		27					11	3		2
**anti-termination**			17				4				
**anti-terminator**			3								
**anti-holin**		18	3								
**anti-recbcd**			2								
**anti-toxin**	1		6					3			
**anti-receptor**									7		
**anti-proliferative**				2							
**anti-immunity**			1								
**anti-dote**			1								

Figure S3. Bar charts are generated from the functional genes associated with each ESKAPE phage species.

(a) Phage genomes infecting *Acinetobacter baumannii*.

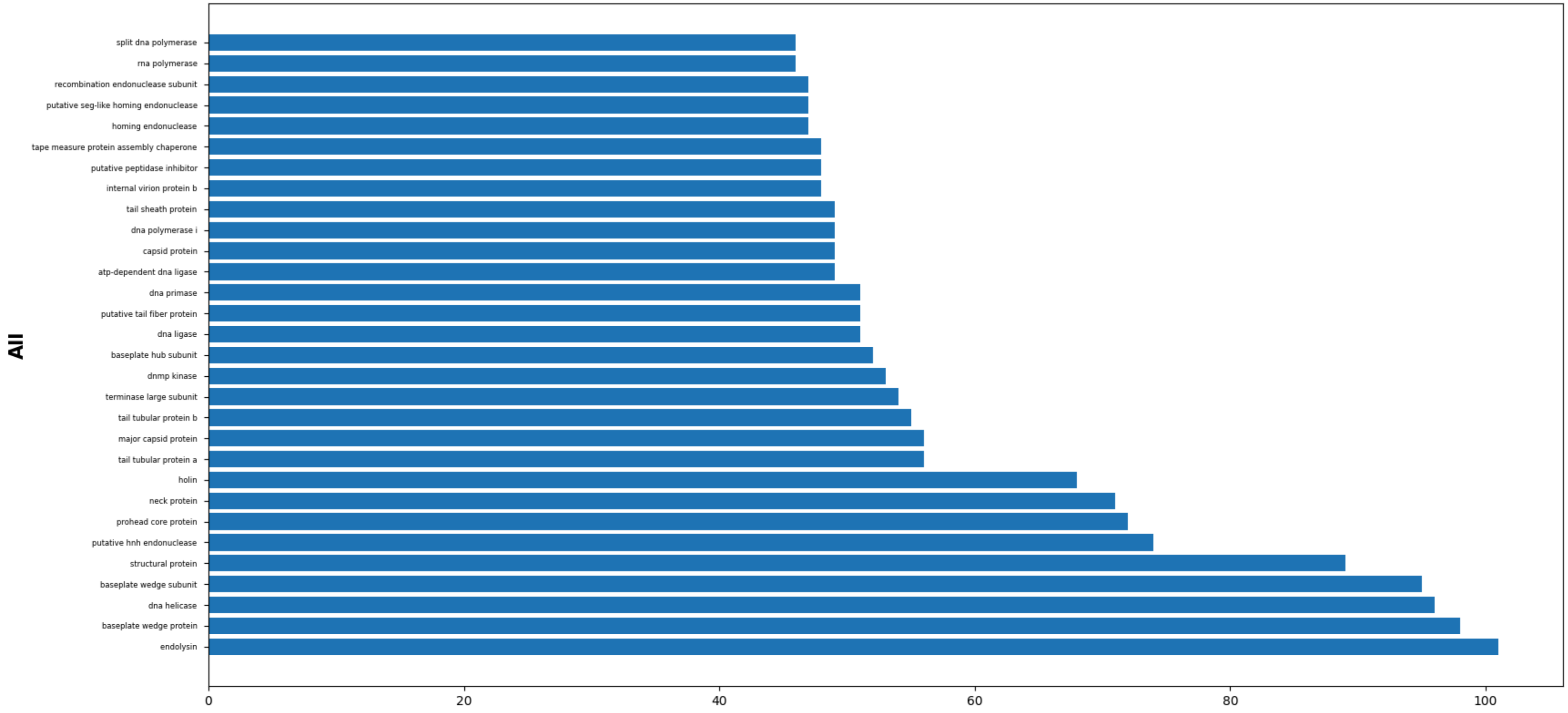

(b) Phage genomes infecting *Campylobacter jejuni.*

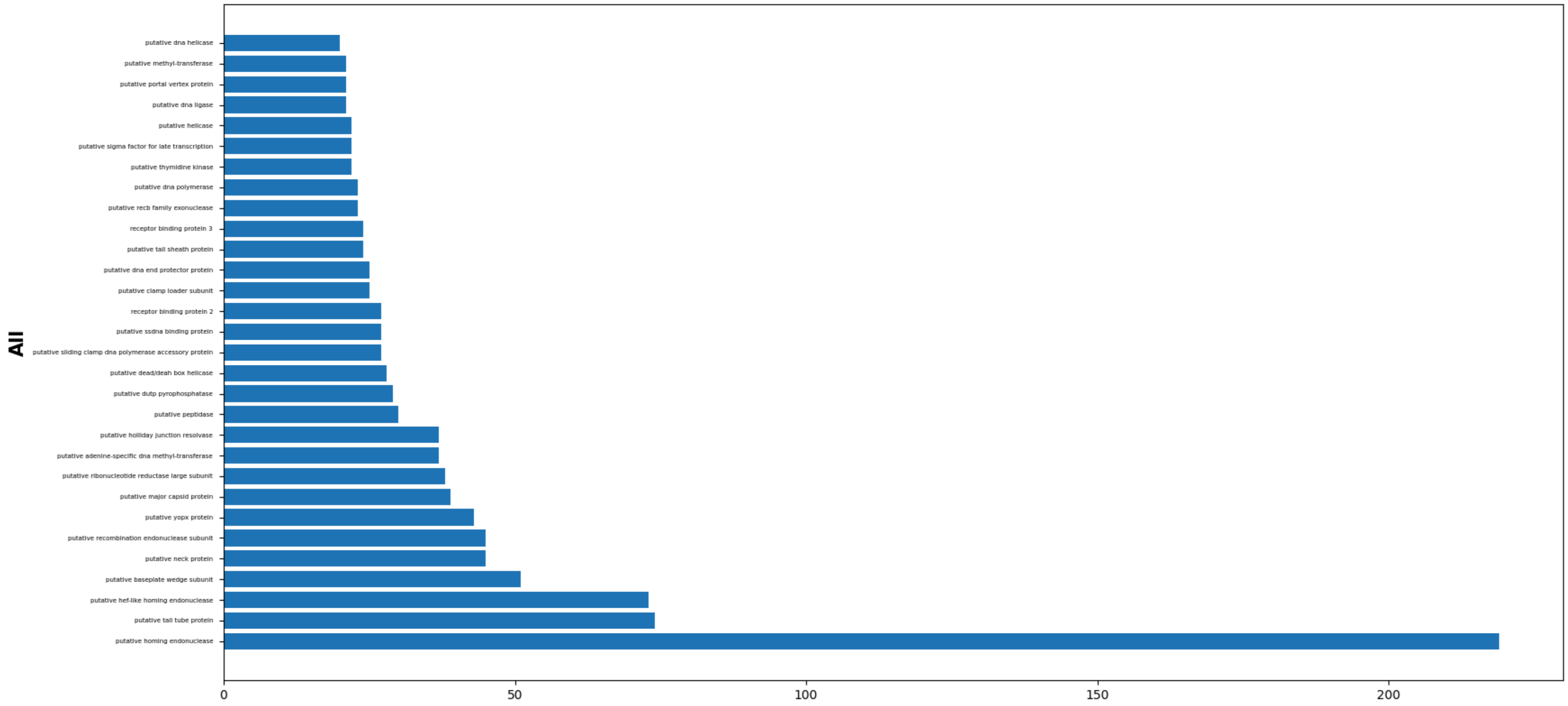

(c) Phage genomes infecting *Escherichia coli*.

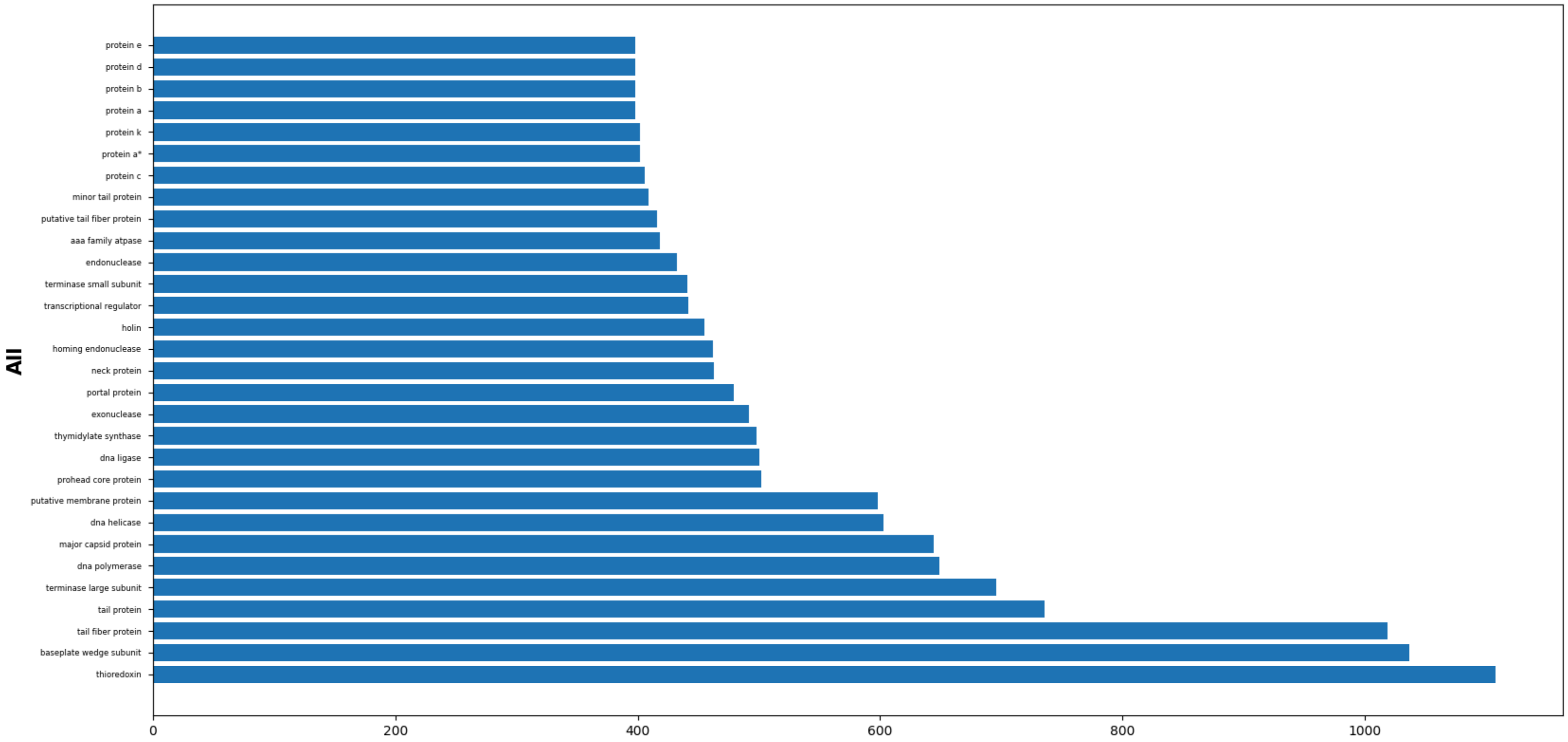

(d) Phage genomes infecting *Enterococcus faecium.*

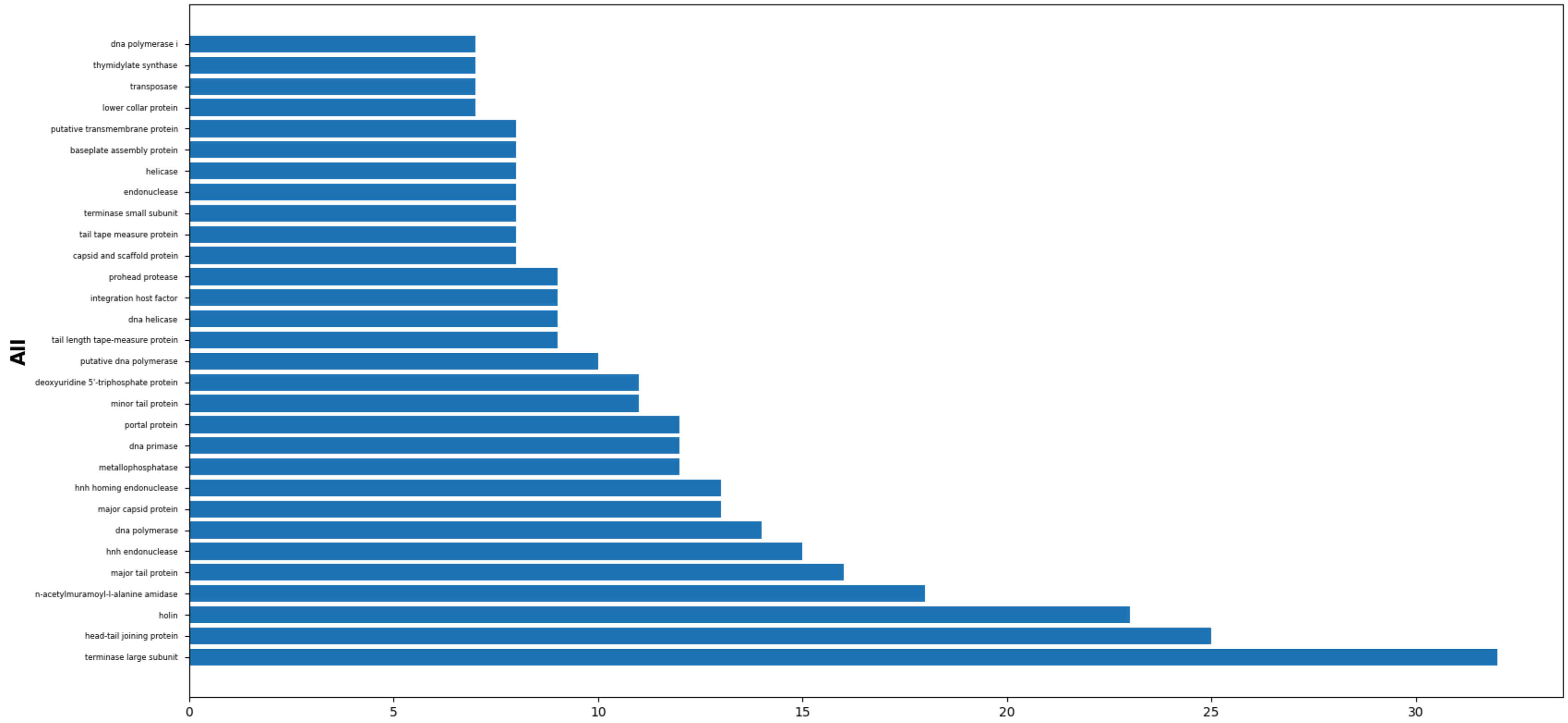

(e) Phage genomes infecting *Haemophilus influenzae*.

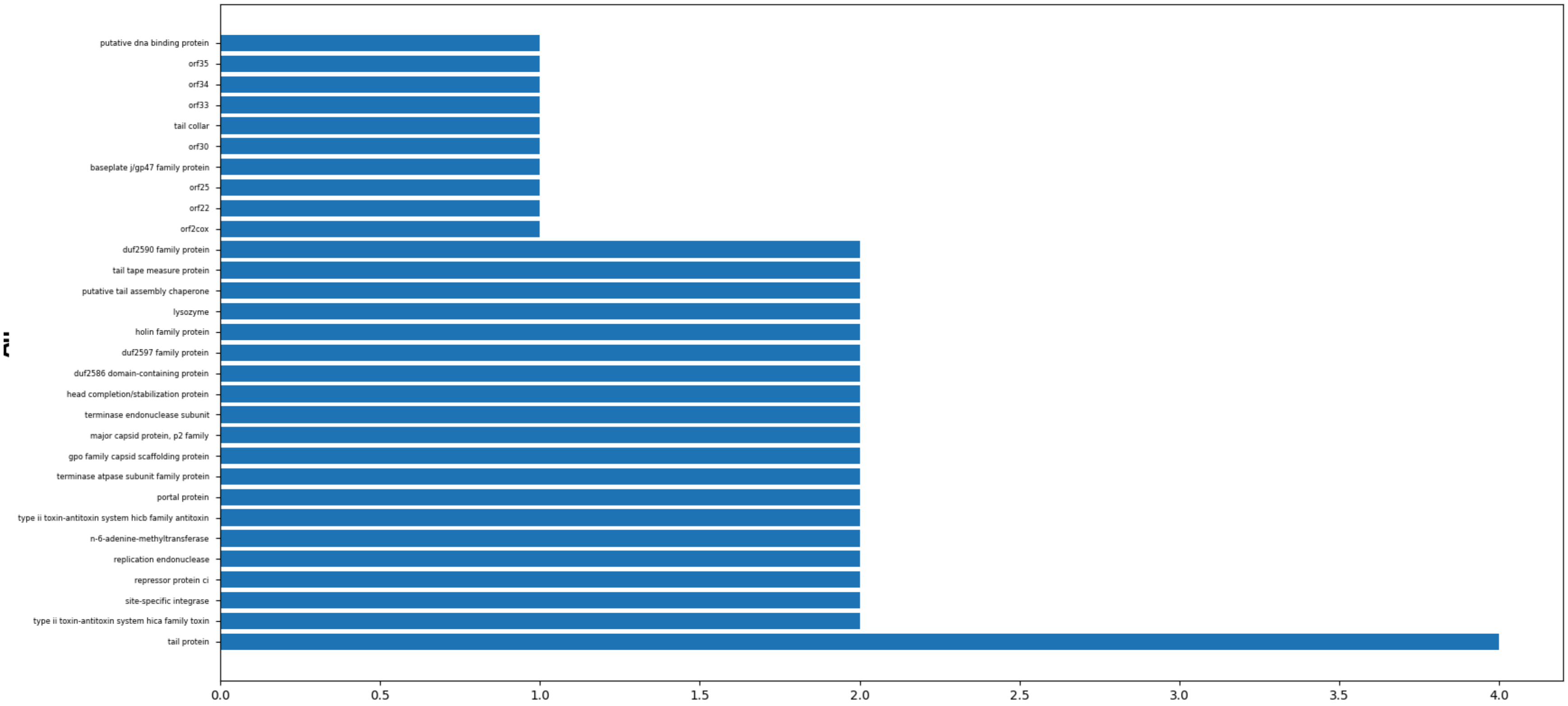

(f) Phage genomes infecting *Helicobacter pylori*.

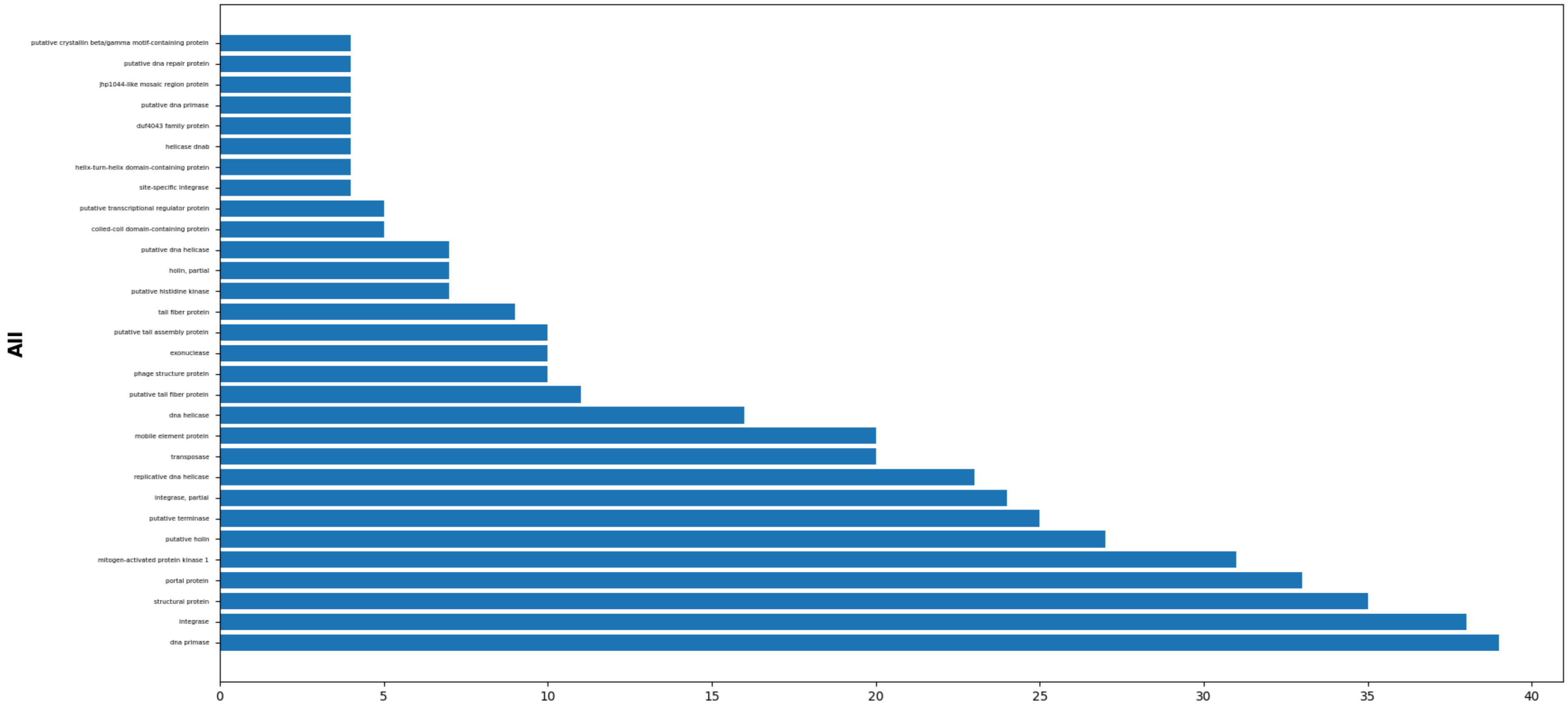

(g) Phage genomes infecting *Pseudomonas aeruginosa*.

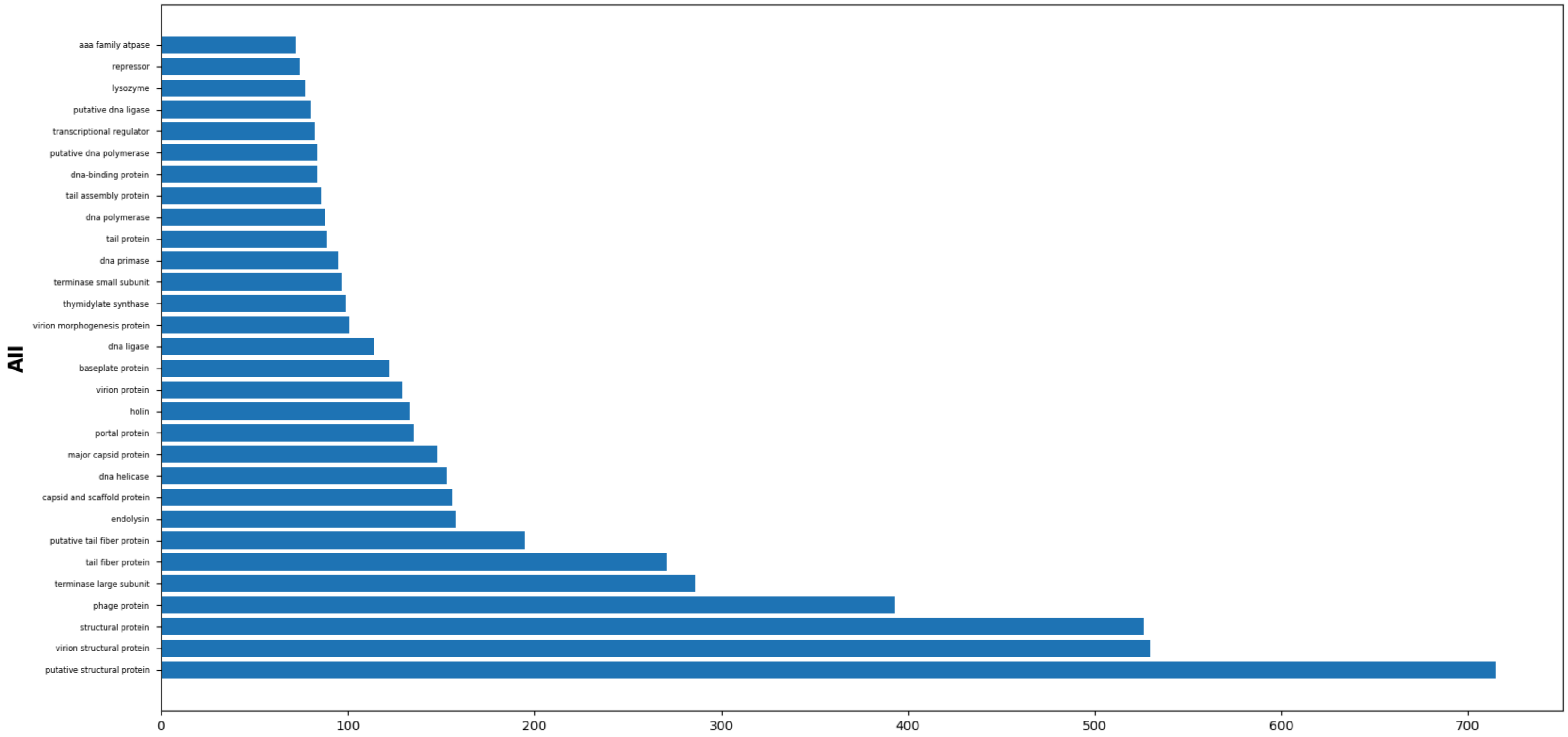

(h) Phage genomes infecting *Staphylococcus aureus.*

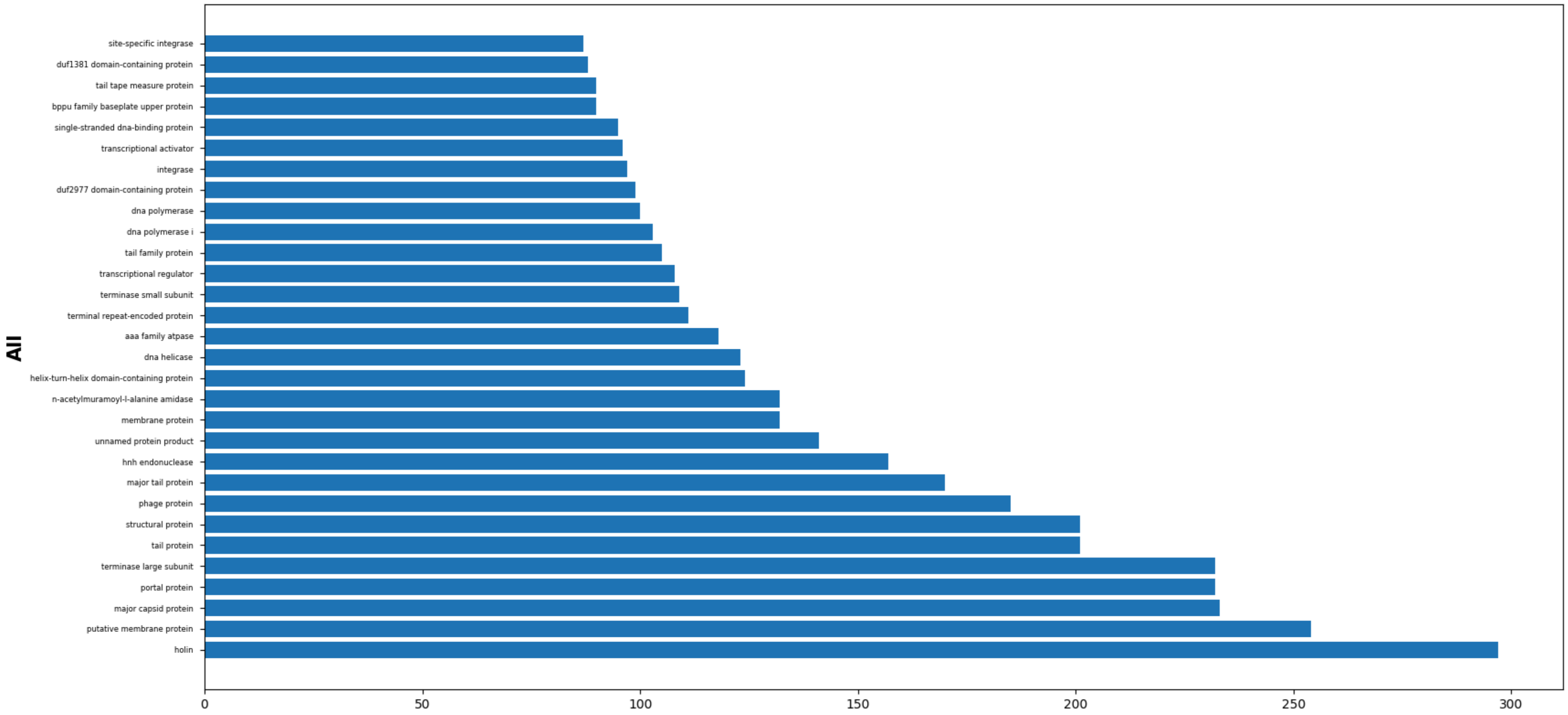

(i) Phage genomes infecting *Streptococcus pneumoniae*.

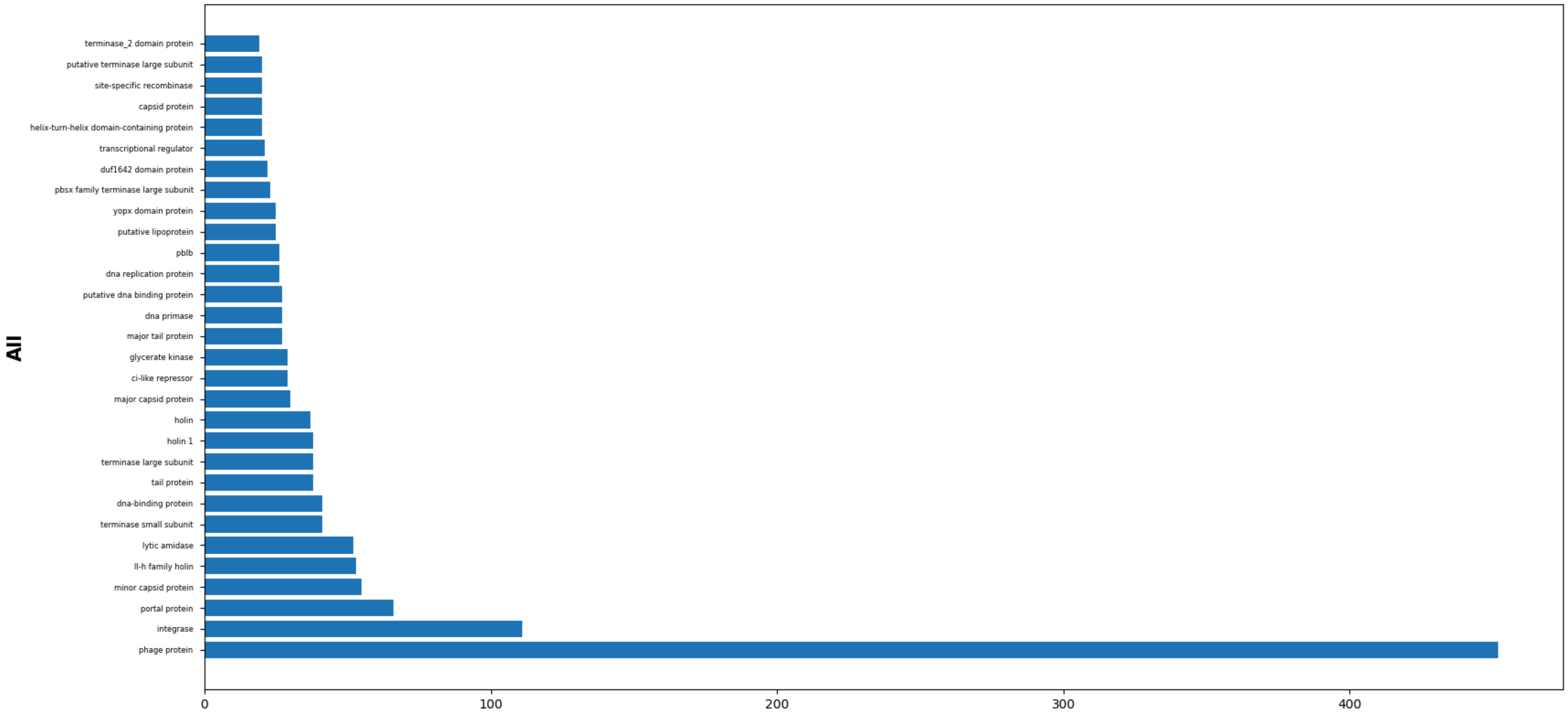

(j) Phage genomes infecting *Salmonella enterica.*

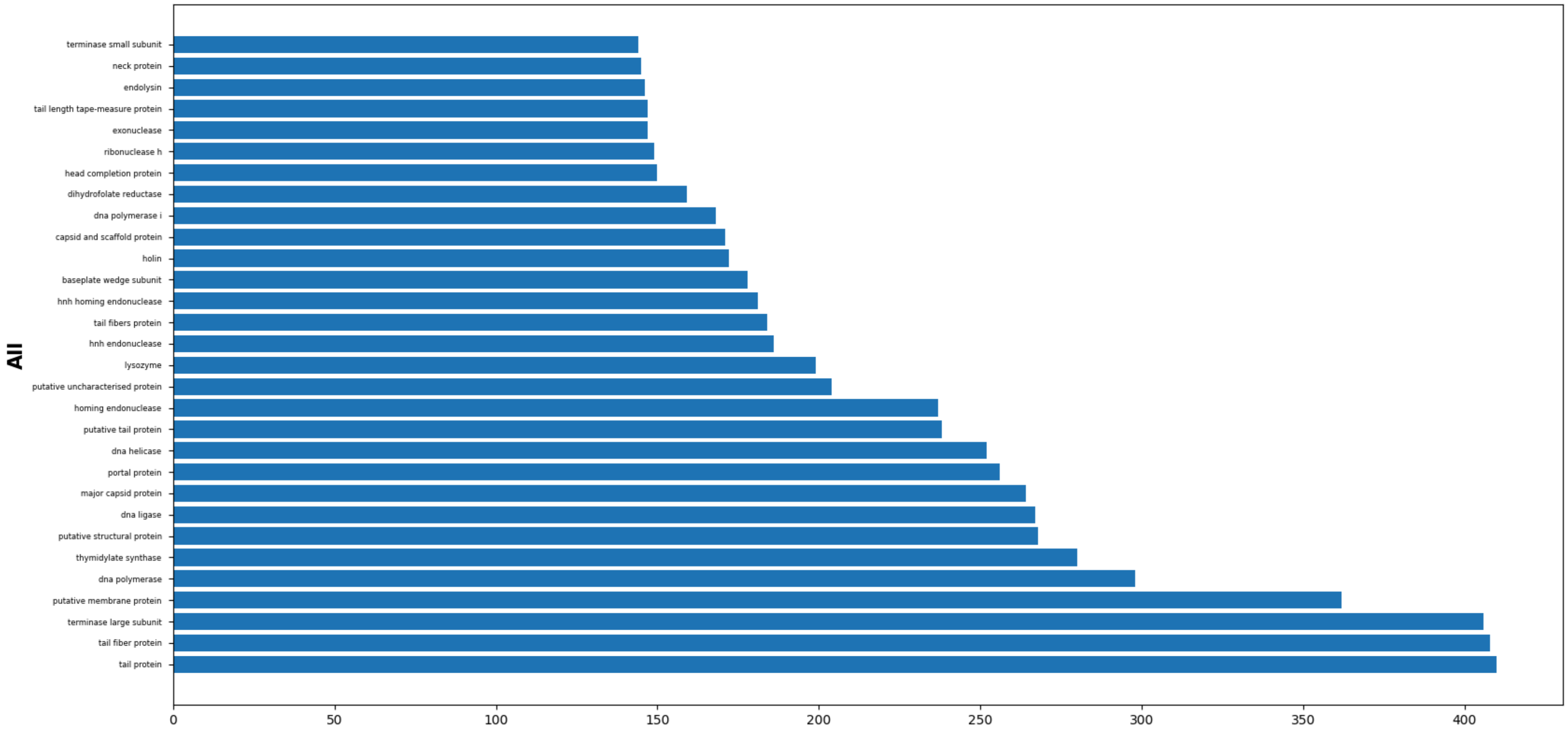

(k) Phage genomes infecting *Shigella flexneri.*

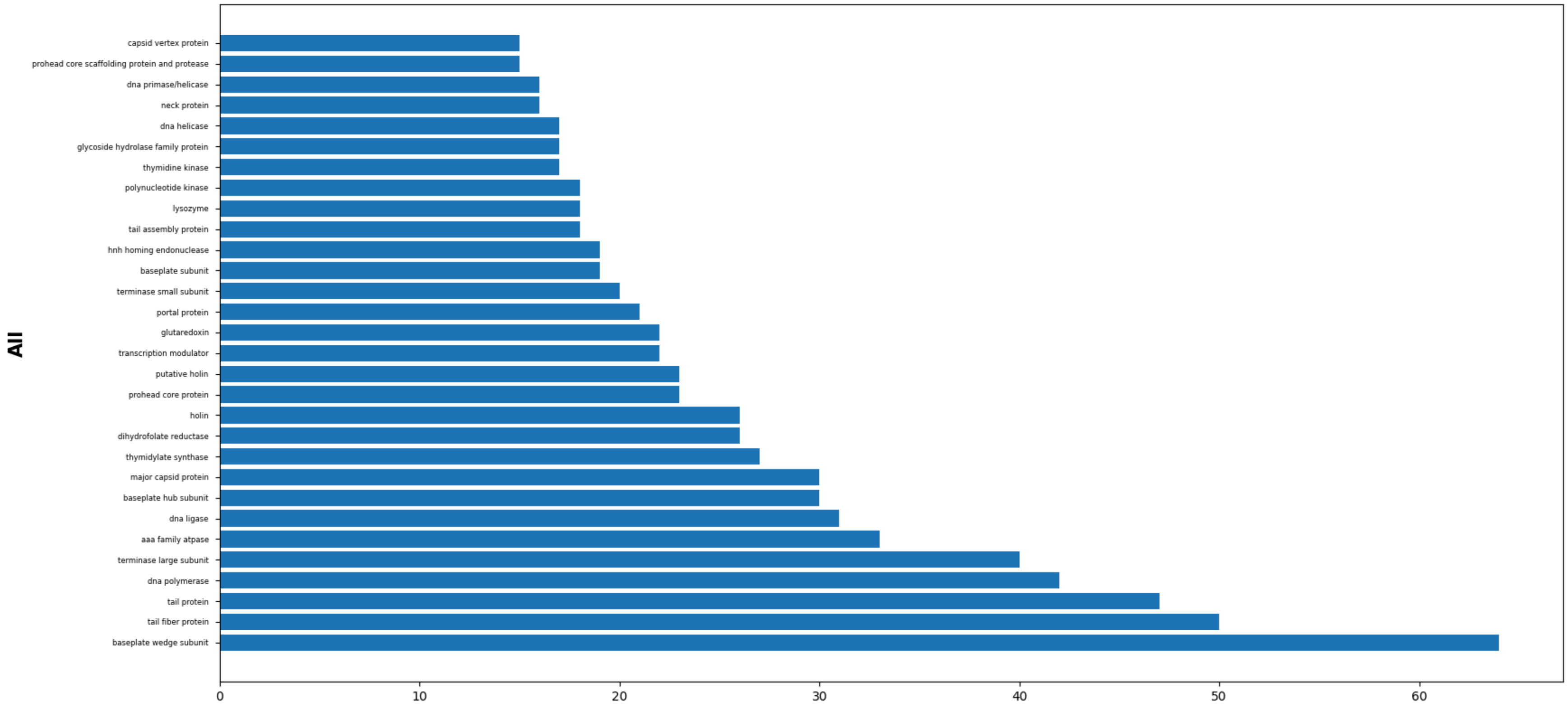

Figure S4. Genomic maps of each ESKAPE phage species.

(a) Representative phage genomes infecting *Haemophilus influenzae*.

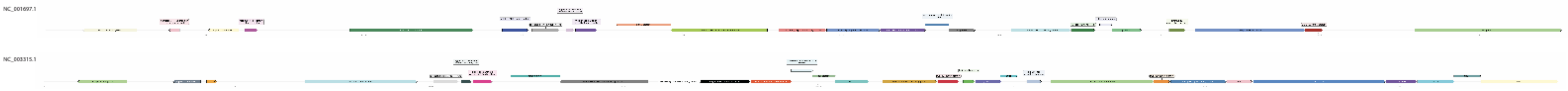

(b) Representative phage genomes infecting *Acinetobacter baumannii*.

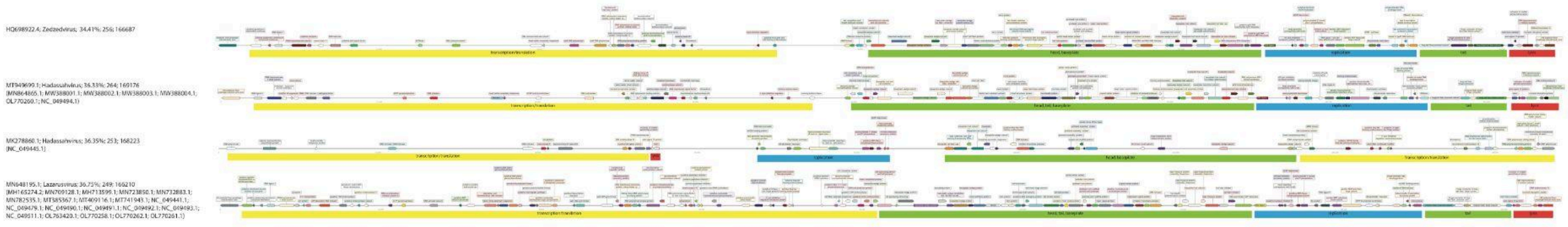

Figure S5. Pangenome analysis of each ESKAPE phage species.

(a) Phage genomes infecting *Acinetobacter baumannii.*

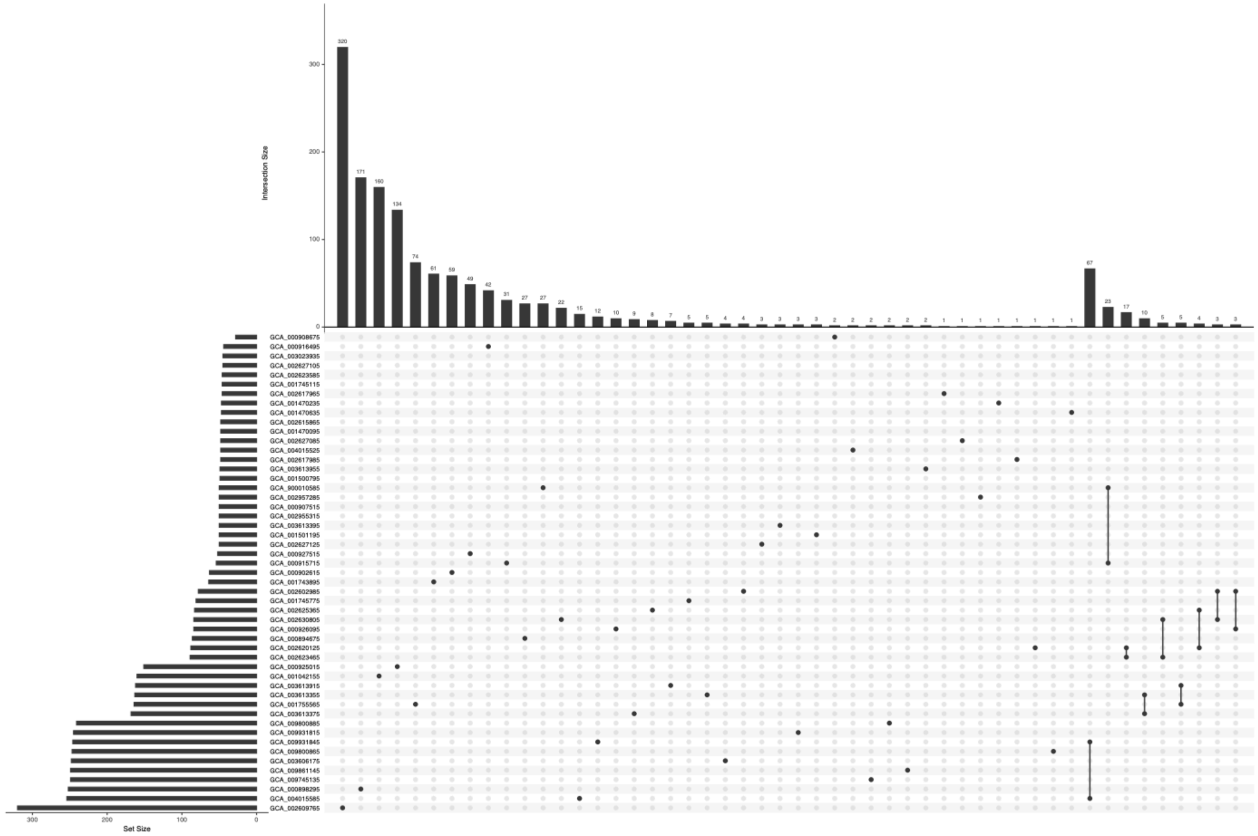

(b) Phage genomes infecting *Campylobacter jejuni*.

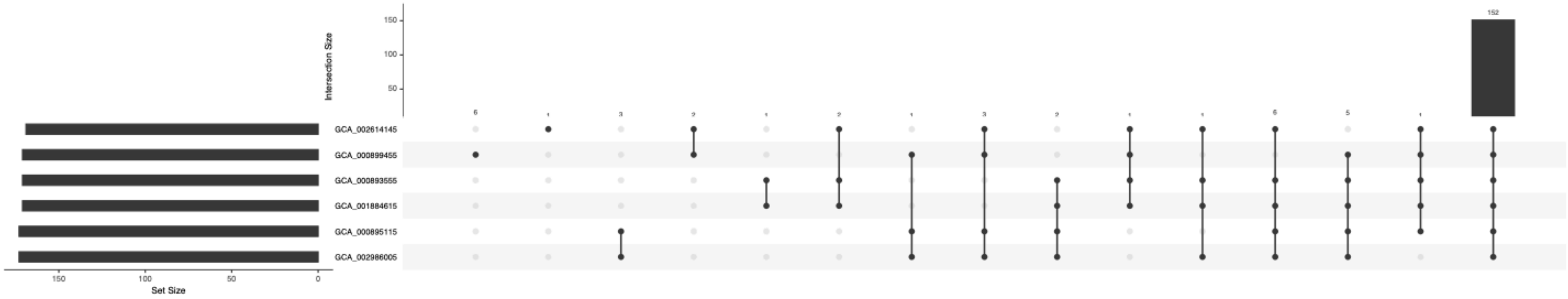

(c) Phage genomes infecting *Escherichia coli*.
N/A

(d) Phage genomes infecting *Enterococcus faecium*.
N/A

(e) Phage genomes infecting *Haemophilus influenzae*.
N/A

(f) Phage genomes infecting *Helicobacter pylori*.

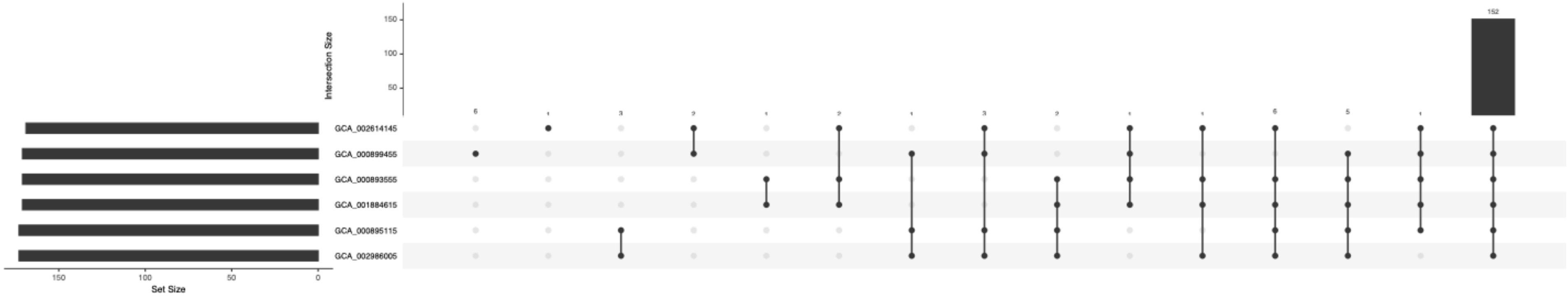

(g) Phage genomes infecting *Pseudomonas aeruginosa*.

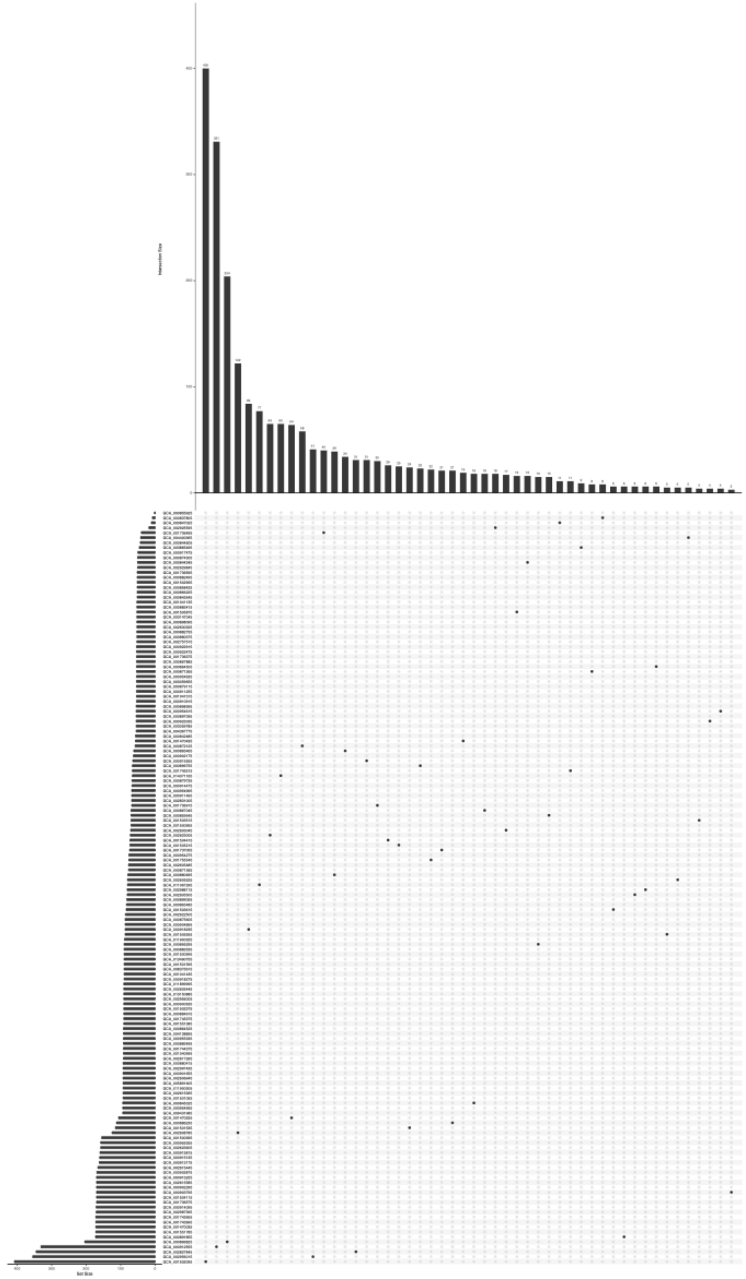

(h) Phage genomes infecting *Staphylococcus aureus*.

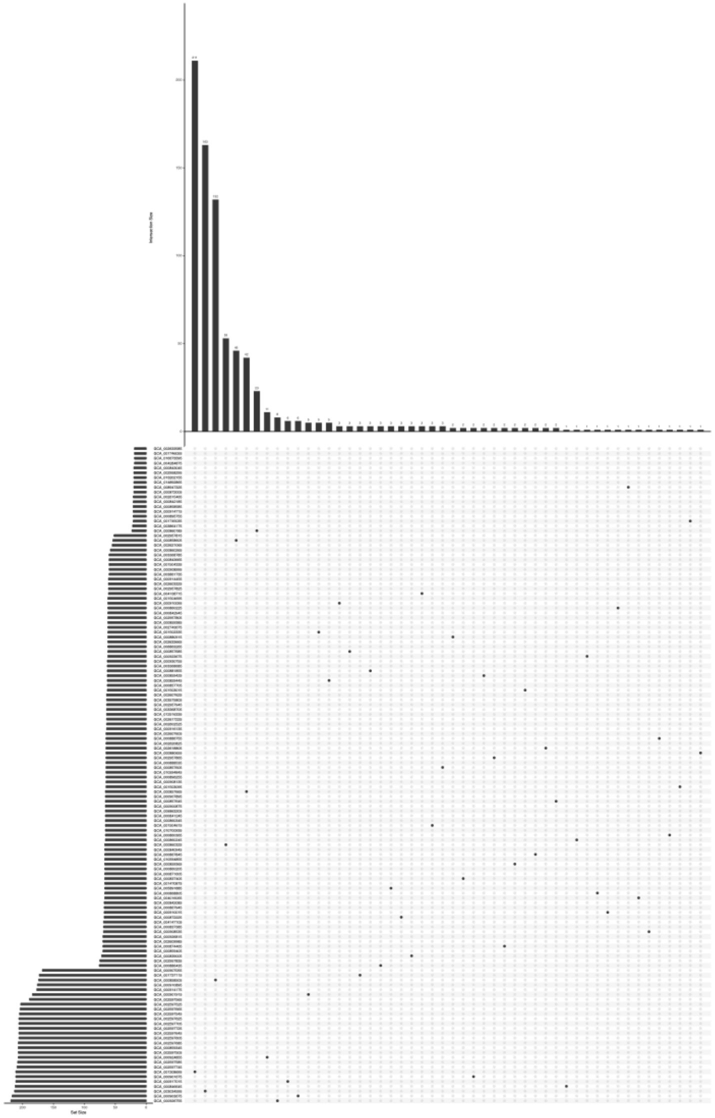

(g) Phage genomes infecting *Streptococcus pneumoniae*.
N/A

(j) Phage genomes infecting *Salmonellae enterica*.

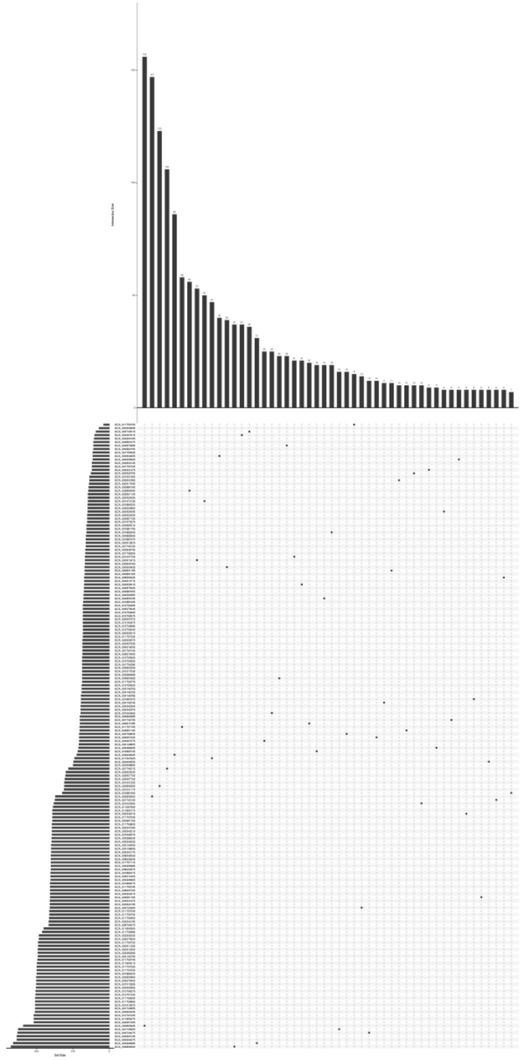

(k) Phage genomes infecting *Shigella flexneri*.

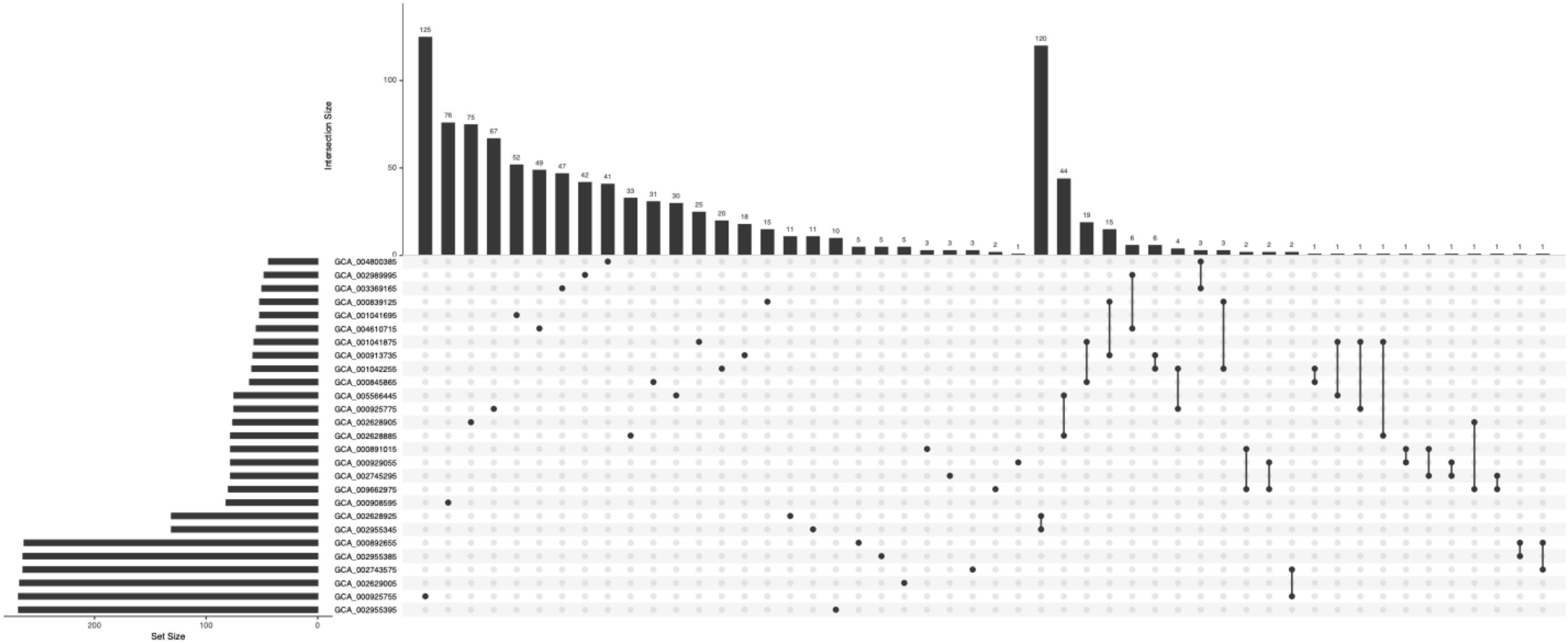

Figure S6. AlphaFold-predicted structures of phage proteins with putative antimicrobial activities.

(a) Representative inhibitor phage proteins.

See *Alphafold_Function.docx* provided.

(b) Representative putative inhibitor phage proteins.

See *Alphafold_Function_putative.docx* provided.

Table S1: Statistics of phage genomes associated with the ESKAPE pathogens.

(a) Phage genomes infecting *Acinetobacter baumannii* (highlighted red are those predicted to be lytic lifestyles with more than 55% probability).
See *ESKAPE_Acinetobacter baumannii.xlsx* provided.

(b) Phage genomes infecting *Campylobacter jejuni* (highlighted red are those predicted to be lytic lifestyles with more than 55% probability).
See *ESKPAE_Campylobacter jejuni.xlsx* provided.

(c) Phage genomes infecting *Escherichia coli* (highlighted red are those predicted to be lytic lifestyles with more than 55% probability).
See *ESKPAE_Escherichia coli.xlsx* provided.

(d) Phage genomes infecting *Enterococcus faecium* (highlighted red are those predicted to be lytic lifestyles with more than 55% probability).
See *ESKAPE_Enterococcus faecium.xlsx* provided.

(e) Phage genomes infecting *Haemophilus influenzae* (highlighted red are those predicted to be lytic lifestyles with more than 55% probability).
See *ESKPAE_Haemophilus influenzae.xlsx* provided.

(f) Phage genomes infecting *Helicobacter pylori* (highlighted red are those predicted to be lytic lifestyles with more than 55% probability).
See *ESKAPE_Helicobacter pylori.xlsx* provided.

(g) Phage genomes infecting *Pseudomonas aeruginosa* (highlighted red are those predicted to be lytic lifestyles with more than 55% probability).
See *ESKPAE_Pseudomonas aeruginosa.xlsx* provided.

(h) Phage genomes infecting *Staphylococcus aureus* (highlighted red are those predicted to be lytic lifestyles with more than 55% probability).
See *ESKAPE_Staphylococcus aureus.xlsx* provided.

(i) Phage genomes infecting *Streptococcus pneumoniae* (highlighted red are those predicted to be lytic lifestyles with more than 55% probability).
See *ESKPAE_Streptococcus pneumoniae.xlsx* provided.

(j) Phage genomes infecting *Salmonellae enterica* (highlighted red are those predicted to be lytic lifestyles with more than 55% probability).
See *ESKAPE_Salmonella enterica.xlsx* provided.

(k) Phage genomes infecting *Shigella flexneri.* (highlighted red are those predicted to be lytic lifestyles with more than 55% probability).
See *ESKAPE_Shigella flexneri.xlsx* provided.

Table S2: Bacteriophages of the ESKAPE pathogens with lytic lifestyles that are potential candidates for phage therapy.

(a) Lytic phage genomes infecting *Acinetobacter baumannii*.

Phage ID	Phage type	DNA type	GC content (%)	Number of protein	Sequence length	Class probability	Phage name
MK278860.1	Hadassahvirus	dsDNA	36.35	253	168223	0.61008991	Ab_Hadaassa_1
NC_049492.1	Lazarusvirus	dsDNA	36.73	252	167922	0.626173826	Ab_Lazarus_1
NC_049493.1	Lazarusvirus	dsDNA	36.76	247	165151	0.618281718	Ab_Lazarus_2
NC_049511.1	Lazarusvirus	dsDNA	36.71	250	166487	0.610889111	Ab_Lazarus_3

(b) Lytic phage genomes infecting *Campylobacter jejuni*.

Phage ID	Phage type	DNA type	GC content (%)	Number of protein	Sequence length	Class probability	Phage name
MT932329.1	Firehammervirus	dsDNA	27.22	211	183102	0.531968032	Cj_Firehammer_1

(c) Lytic phage genomes infecting *Escherichia coli*.

Phage ID	Phage type	DNA type	GC content (%)	Number of protein	Sequence length	Class probability	Phage name
AP011113.1	Tequatrovirus	dsDNA	35.33	0	167435	0.792807193	Ec_Tequatro_1
EU863409.1	Dhakavirus	dsDNA	39.52	3	171451	0.798901099	Ec_Dhaka_1
HM137666.1	Tequatrovirus	dsDNA	35.29	4	168920	0.810989011	Ec_Tequatro_2
HM997020.1	Tequatrovirus	dsDNA	35.4	11	166452	0.808691309	Ec_Tequatro_3
JN202312.1	Siphoviridae	dsDNA	35.67	11	166499	0.794205794	Ec_Sipho_1
JX128259.1	Tequatrovirus	dsDNA	35.41	0	166783	0.781318681	Ec_Tequatro_4
JX421753.1	Teetrevirus	dsDNA	49.91	0	38202	0.806193806	Ec_Teetre_1
KM606994.1	Tequatrovirus	dsDNA	35.37	11	168402	0.829270729	Ec_Tequatro_5
KM606996.1	Tequatrovirus	dsDNA	35.37	11	168394	0.816883117	Ec_Tequatro_6
KM606997.1	Tequatrovirus	dsDNA	35.37	11	168395	0.812487512	Ec_Tequatro_7
KM606998.1	Tequatrovirus	dsDNA	35.37	11	168395	0.81028971	Ec_Tequatro_8
KM606999.1	Tequatrovirus	dsDNA	35.37	11	168401	0.808691309	Ec_Tequatro_9
KM607000.1	Tequatrovirus	dsDNA	35.49	11	165179	0.818581419	Ec_Tequatro_10

KM607001.1	Tequatrovirus	dsDNA	35.33	11	166007	0.857742258	Ec_Tequatro_11
KM607002.1	Tequatrovirus	dsDNA	35.29	11	168896	0.814885115	Ec_Tequatro_12
KM607003.1	Tequatrovirus	dsDNA	35.29	11	168966	0.810589411	Ec_Tequatro_13
KM607004.1	Tequatrovirus	dsDNA	35.25	11	168401	0.844055944	Ec_Tequatro_14
KP869105.1	Tequatrovirus	dsDNA	35.4	0	167747	0.822977023	Ec_Tequatro_15
KR233165.1	Tequatrovirus	dsDNA	35.47	11	167552	0.802397602	Ec_Tequatro_16
KT184311.1	Tequatrovirus	dsDNA	35.45	11	166514	0.793306693	Ec_Tequatro_17
KT184312.1	Tequatrovirus	dsDNA	35.55	11	167318	0.793206793	Ec_Tequatro_18
KU867876.1	Tequatrovirus	dsDNA	35.54	11	165772	0.789210789	Ec_Tequatro_19
KU925172.1	Tequatrovirus	dsDNA	35.35	11	166423	0.812487512	Ec_Tequatro_20
KX009778.3	Tequatrovirus	dsDNA	35.28	11	170788	0.803596404	Ec_Tequatro_21
LN881726.1	Tequatrovirus	dsDNA	35.52	16	167298	0.792407592	Ec_Tequatro_22
LN881728.1	Tequatrovirus	dsDNA	35.4	50	167467	0.791008991	Ec_Tequatro_23
LN881733.1	Tequatrovirus	dsDNA	35.4	25	167467	0.782117882	Ec_Tequatro_24
LN881734.1	Tequatrovirus	dsDNA	35.51	28	167298	0.781818182	Ec_Tequatro_25
LN881737.1	Tequatrovirus	dsDNA	35.51	48	167299	0.791508492	Ec_Tequatro_26
MF001359.1	Tequatrovirus	dsDNA	35.53	56	168805	0.793906094	Ec_Tequatro_27
MF001360.1	Tequatrovirus	dsDNA	35.47	50	168638	0.8001998	Ec_Tequatro_28
MG781191.1	Tequatrovirus	dsDNA	35.33	55	167064	0.793606394	Ec_Tequatro_29
MG833025.1	Tequatrovirus	dsDNA	48.39	54	38553	0.791908092	Ec_Tequatro_30
MH051915.1	Tequatrovirus	dsDNA	35.64	53	164366	0.782417582	Ec_Tequatro_31
MH243439.1	Tequatrovirus	dsDNA	35.45	61	166083	0.792807193	Ec_Tequatro_32
MH550421.1	Tequatrovirus	dsDNA	35.22	60	168706	0.780519481	Ec_Tequatro_33
MH553563.1	Tequatrovirus	dsDNA	35.37	54	166677	0.828671329	Ec_Tequatro_34
MH751506.1	Tequatrovirus	dsDNA	35.32	74	163832	0.802097902	Ec_Tequatro_35
MH992121.1	Tequatrovirus	dsDNA	35.39	59	167379	0.813586414	Ec_Tequatro_36
MH992510.1	Tequatrovirus	dsDNA	35.4	66	166040	0.800799201	Ec_Tequatro_37
MK327929.1	Tequatrovirus	dsDNA	35.39	71	168049	0.802797203	Ec_Tequatro_38
MK327936.1	Tequatrovirus	dsDNA	35.36	69	168886	0.781718282	Ec_Tequatro_39

MK327937.1	Tequatrovirus	dsDNA	35.29	69	170182	0.793806194	Ec_Tequatro_40
MK327939.1	Tequatrovirus	dsDNA	35.46	63	167826	0.785214785	Ec_Tequatro_41
MK327942.1	Tequatrovirus	dsDNA	35.52	60	167728	0.801798202	Ec_Tequatro_42
MK327945.1	Tequatrovirus	dsDNA	35.32	76	168363	0.807392607	Ec_Tequatro_43
MK327946.1	Tequatrovirus	dsDNA	35.37	75	168828	0.781318681	Ec_Tequatro_44
MK327947.1	Krischvirus	dsDNA	40.44	71	164278	0.784015984	Ec_Krisch_1
MK373781.1	Tequatrovirus	dsDNA	35.37	66	164987	0.800699301	Ec_Tequatro_45
MK373785.1	Tequatrovirus	dsDNA	35.22	76	168756	0.8000999	Ec_Tequatro_46
MK962750.1	Tequatrovirus	dsDNA	35.28	71	167247	0.782617383	Ec_Tequatro_47
MK962752.1	Tequatrovirus	dsDNA	35.32	78	168349	0.787712288	Ec_Tequatro_48
MK962755.1	Tequatrovirus	dsDNA	35.48	78	167852	0.793106893	Ec_Tequatro_49
MK977694.1	Tequatrovirus	dsDNA	35.35	75	167353	0.824675325	Ec_Tequatro_50
MN022785.1	Tequatrovirus	dsDNA	35.46	84	167532	0.787012987	Ec_Tequatro_51
MN508616.2	Tequatrovirus	dsDNA	35.38	76	166781	0.788611389	Ec_Tequatro_52
MN895434.1	Tequatrovirus	dsDNA	35.33	93	167892	0.786613387	Ec_Tequatro_53
MN895438.1	Tequatrovirus	dsDNA	35.44	93	166833	0.818681319	Ec_Tequatro_54
MT764206.1	Tequatrovirus	dsDNA	35.31	133	170340	0.78011988	Ec_Tequatro_55
MT884007.2	Tequatrovirus	dsDNA	35.4	123	166364	0.781518482	Ec_Tequatro_56
MT932213.1	Tequatrovirus	dsDNA	35.43	119	166100	0.796803197	Ec_Tequatro_57
MT984581.1	Tequatrovirus	dsDNA	35.29	115	168908	0.79000999	Ec_Tequatro_58
MW286157.1	Tequatrovirus	dsDNA	35.37	133	165973	0.801598402	Ec_Tequatro_59
MW749001.1	Tequatrovirus	dsDNA	35.61	113	167034	0.786013986	Ec_Tequatro_60
MZ065353.1	Tequatrovirus	dsDNA	35.55	126	167440	0.813086913	Ec_Tequatro_61
MZ170041.1	Tequatrovirus	dsDNA	35.36	143	168411	0.793406593	Ec_Tequatro_62
MZ189262.1	Tequatrovirus	dsDNA	35.46	124	166244	0.788911089	Ec_Tequatro_63
MZ234013.1	Tequatrovirus	dsDNA	35.22	167	169555	0.797502498	Ec_Tequatro_64
MZ234031.1	Tequatrovirus	dsDNA	35.45	165	167922	0.782217782	Ec_Tequatro_65
MZ234034.1	Tequatrovirus	dsDNA	35.37	170	168524	0.784615385	Ec_Tequatro_66
MZ234039.1	Tequatrovirus	dsDNA	35.37	170	168522	0.781118881	Ec_Tequatro_67

MZ234042.1	Tequatrovirus	dsDNA	35.36	168	169416	0.817782218	Ec_Tequatro_68
MZ234051.1	Tequatrovirus	dsDNA	35.37	233	169415	0.781418581	Ec_Tequatro_69
MZ234052.1	Tequatrovirus	dsDNA	35.36	218	169292	0.787312687	Ec_Tequatro_70
MZ234053.1	Tequatrovirus	dsDNA	35.33	198	169519	0.792507493	Ec_Tequatro_71
MZ291552.2	Tequatrovirus	dsDNA	35.42	220	165859	0.801698302	Ec_Tequatro_72
MZ501050.1	Tequatrovirus	dsDNA	35.46	213	168255	0.803496503	Ec_Tequatro_73
MZ501066.1	Tequatrovirus	dsDNA	35.45	179	169509	0.805594406	Ec_Tequatro_74
MZ501067.1	Tequatrovirus	dsDNA	35.42	223	165574	0.804095904	Ec_Tequatro_75
MZ501089.1	Tequatrovirus	dsDNA	35.37	238	167832	0.804595405	Ec_Tequatro_76
MZ501096.1	Tequatrovirus	dsDNA	35.41	240	167732	0.788911089	Ec_Tequatro_77
MZ501098.1	Tequatrovirus	dsDNA	35.64	216	170053	0.787612388	Ec_Tequatro_78
MZ501106.1	Tequatrovirus	dsDNA	35.43	249	166115	0.793506494	Ec_Tequatro_79
MZ501113.1	Tequatrovirus	dsDNA	35.44	239	166861	0.794805195	Ec_Tequatro_80
MZ502380.1	Tequatrovirus	dsDNA	35.53	212	165679	0.781718282	Ec_Tequatro_81
MZ681930.1	Tequatrovirus	dsDNA	35.62	281	164663	0.78001998	Ec_Tequatro_82
MZ726795.1	Tequatrovirus	dsDNA	35.37	273	166374	0.81978022	Ec_Tequatro_83
MZ753803.1	Tequatrovirus	dsDNA	35.42	275	166986	0.788811189	Ec_Tequatro_84
NC_000866.4	Tequatrovirus	dsDNA	35.3	276	168903	0.81988012	Ec_Tequatro_85
NC_005066.1	Krischvirus	dsDNA	40.44	272	164018	0.799200799	Ec_Krisch_2
NC_009821.1	Krischvirus	dsDNA	40.5	268	164270	0.841458541	Ec_Krisch_3
NC_010105.1	Dhakavirus	dsDNA	39.51	267	170523	0.784015984	Ec_Dhaka_2
NC_012740.1	Krischvirus	dsDNA	40.51	271	166418	0.814085914	Ec_Krisch_4
NC_012741.1	Dhakavirus	dsDNA	39.52	268	171451	0.845854146	Ec_Dhaka_3
NC_019399.1	Tequatrovirus	dsDNA	35.24	274	167396	0.783116883	Ec_Tequatro_86
NC_019505.1	Tequatrovirus	dsDNA	35.4	267	166452	0.810689311	Ec_Tequatro_87
NC_024125.2	Tequatrovirus	dsDNA	35.28	274	168470	0.78961039	Ec_Tequatro_88
NC_025419.1	Tequatrovirus	dsDNA	35.37	273	168402	0.826873127	Ec_Tequatro_89
NC_025448.1	Tequatrovirus	dsDNA	35.49	285	165179	0.807892108	Ec_Tequatro_90
NC_025449.1	Tequatrovirus	dsDNA	35.41	279	166783	0.813886114	Ec_Tequatro_91

NC_027979.1	Tequatrovirus	dsDNA	35.25	267	168401	0.816183816	Ec_Tequatro_92
NC_027983.1	Tequatrovirus	dsDNA	35.33	269	167435	0.808791209	Ec_Tequatro_93
NC_031030.1	Tequatrovirus	dsDNA	35.28	277	170788	0.801998002	Ec_Tequatro_94
NC_047867.1	Teetrevirus	dsDNA	49.91	515	38202	0.803196803	Ec_Teetre_2
NC_054902.1	Tequatrovirus	dsDNA	35.53	58	168496	0.796903097	Ec_Tequatro_95
NC_054904.1	Tequatrovirus	dsDNA	35.55	67	165549	0.784315684	Ec_Tequatro_96
NC_054906.1	Tequatrovirus	dsDNA	35.37	67	166677	0.787212787	Ec_Tequatro_97
NC_054908.1	Tequatrovirus	dsDNA	35.4	59	166040	0.785614386	Ec_Tequatro_98
NC_054911.1	Tequatrovirus	dsDNA	35.47	52	168638	0.795604396	Ec_Tequatro_99
NC_054914.1	Tequatrovirus	dsDNA	35.34	60	167076	0.789410589	Ec_Tequatro_100
NC_054916.1	Tequatrovirus	dsDNA	35.35	53	170121	0.794305694	Ec_Tequatro_101
NC_054918.1	Tequatrovirus	dsDNA	35.46	57	167826	0.789210789	Ec_Tequatro_102
NC_054919.1	Tequatrovirus	dsDNA	35.37	51	168828	0.790609391	Ec_Tequatro_103
NC_054920.1	Tequatrovirus	dsDNA	35.3	60	168670	0.788211788	Ec_Tequatro_104
NC_054922.1	Tequatrovirus	dsDNA	35.37	63	164987	0.78021978	Ec_Tequatro_105
NC_054923.1	Tequatrovirus	dsDNA	35.34	71	166848	0.8002997	Ec_Tequatro_106
NC_054925.1	Tequatrovirus	dsDNA	35.45	56	166083	0.782517483	Ec_Tequatro_107
NC_054926.1	Tequatrovirus	dsDNA	35.22	60	168756	0.811488511	Ec_Tequatro_108
NC_054932.1	Tequatrovirus	dsDNA	35.44	55	166833	0.799200799	Ec_Tequatro_109
NC_054933.1	Tequatrovirus	dsDNA	35.33	62	167892	0.810589411	Ec_Tequatro_110
NC_055741.1	Tequatrovirus	dsDNA	35.64	56	164366	0.78991009	Ec_Tequatro_111
NC_055780.1	Tequatrovirus	dsDNA	35.39	67	167379	0.81038961	Ec_Tequatro_112
OK040907.1	Tequatrovirus	dsDNA	36	74	168395	0.789210789	Ec_Tequatro_113
OK076929.1	Tequatrovirus	dsDNA	35.34	62	167069	0.793006993	Ec_Tequatro_114
OK272476.1	Tequatrovirus	dsDNA	35.52	74	168125	0.786113886	Ec_Tequatro_115
OK272484.1	Tequatrovirus	dsDNA	35.49	76	166300	0.788811189	Ec_Tequatro_116
OL362041.1	Tequatrovirus	dsDNA	35.32	79	169246	0.811588412	Ec_Tequatro_117
OL770073.1	Tequatrovirus	dsDNA	35.55	72	167390	0.786313686	Ec_Tequatro_118
OL870316.1	Tequatrovirus	dsDNA	35.43	79	166367	0.826973027	Ec_Tequatro_119

OL960580.1	Tequatrovirus	dsDNA	35.64	84	167177	0.790809191	Ec_Tequatro_120
OL960581.1	Tequatrovirus	dsDNA	35.46	91	166337	0.784115884	Ec_Tequatro_121
OM135583.1	Tequatrovirus	dsDNA	35.44	90	165577	0.787912088	Ec_Tequatro_122
OM386666.1	Tequatrovirus	dsDNA	35.28	110	167844	0.803096903	Ec_Tequatro_123
ON210139.1	Tequatrovirus	dsDNA	35.35	256	171798	0.790909091	Ec_Tequatro_124
ON645936.1	Tequatrovirus	dsDNA	35.33	273	169736	0.796103896	Ec_Tequatro_125
ON782582.1	unknown	unknown	35.43	277	166195	0.795204795	Ec_unknown_1
OP168898.1	Tequatrovirus	dsDNA	35.43	269	165622	0.792607393	Ec_Tequatro_126

(d) Lytic phage genomes infecting *Enterococcus faecium*.

**Phage ID**	**Phage type**	**DNA type**	**GC content (%)**	**Number of protein**	**Sequence length**	**Class probability**	**Phage name**
LC606223.1	Siphoviridae	dsDNA	54.17	1	480	0.530769231	Ef_Sipho_1

(e) Lytic phage genomes infecting *Helicobacter pylori*.

**Phage ID**	**Phage type**	**DNA type**	**GC content (%)**	**Number of protein**	**Sequence length**	**Class probability**	**Phage name**
MW160243.1	Schmidvirus	dsDNA	37.82	1	193	0.542257742	Hp_Schmid_1

(f) Lytic phage genomes infecting *Pseudomonas aeruginosa*.

**Phage ID**	**Phage type**	**DNA type**	**GC content (%)**	**Number of protein**	**Sequence length**	**Class probability**	**Phage name**
AM910650.1	Bruynoghevirus	dsDNA	52.25	68	45625	0.646853147	Pa_Bruynoghe_1
AM910651.1	Phikmvvirus	dsDNA	62.28	54	43548	0.612287712	Pa_Phikmv_1
EU056923.1	Phikmvvirus	dsDNA	62.33	52	42954	0.61038961	Pa_Phikmv_2
FN263372.1	Phikmvvirus	dsDNA	62.87	53	43152	0.723676324	Pa_Phikmv_3
JQ307386.1	Phikmvvirus	dsDNA	62.27	51	42966	0.606793207	Pa_Phikmv_4
JX501340.1	Phikmvvirus	dsDNA	62.14	53	42874	0.588411588	Pa_Phikmv_5
JX997978.1	Phikmvvirus	dsDNA	62.28	50	42957	0.593006993	Pa_Phikmv_6
KR054031.1	Phikmvvirus	dsDNA	62.2	55	42508	0.582717283	Pa_Phikmv_7
KU743887.1	Phikmvvirus	dsDNA	62.26	49	42351	0.64025974	Pa_Phikmv_8

KX711710.1	Phikmvvirus	dsDNA	62.26	54	42965	0.61968032	Pa_Phikmv_9
KY618819.1	Phikmvvirus	dsDNA	62.29	60	43337	0.611688312	Pa_Phikmv_10
LN610574.1	Phikmvvirus	dsDNA	62.35	54	43639	0.592807193	Pa_Phikmv_11
M11912.1	Tertilicivirus	dsDNA	45.36	9	5833	0.65014985	Pa_Tertilici_1
M19377.1	Tertilicivirus	dsDNA	45.38	9	5833	0.639460539	Pa_Tertilici_2
MH107770.1	Phikmvvirus	dsDNA	62.36	62	44205	0.591208791	Pa_Phikmv_12
MN602045.1	Phikmvvirus	dsDNA	62.16	54	42735	0.595404595	Pa_Phikmv_13
MN615698.1	Phikmvvirus	dsDNA	62.35	56	43125	0.585314685	Pa_Phikmv_14
MN615699.1	Phikmvvirus	dsDNA	62.25	53	42410	0.601298701	Pa_Phikmv_15
MN692672.2	Phikmvvirus	dsDNA	62.16	54	42735	0.623376623	Pa_Phikmv_16
MN901924.1	Phikmvvirus	dsDNA	62.3	55	43583	0.597102897	Pa_Phikmv_17
MW117144.1	Phikmvvirus	dsDNA	62.29	48	40977	0.588011988	Pa_Phikmv_18
MW406975.1	Phikmvvirus	dsDNA	62.21	52	43005	0.6	Pa_Phikmv_19
MW406976.1	Phikmvvirus	dsDNA	62.24	53	43113	0.610789211	Pa_Phikmv_20
MZ553931.1	Phikmvvirus	dsDNA	62.29	52	42546	0.613486513	Pa_Phikmv_21
NC_001418.1	Tertilicivirus	dsDNA	45.36	9	5833	0.591908092	Pa_Tertilici_3
NC_005045.1	Phikmvvirus	dsDNA	62.3	49	42519	0.716683317	Pa_Phikmv_22
NC_010325.1	Bruynoghevirus	dsDNA	52.25	68	45625	0.605594406	Pa_Bruynoghe_2
NC_011105.1	Phikmvvirus	dsDNA	62.33	52	42954	0.652247752	Pa_Phikmv_23
NC_011107.1	Phikmvvirus	dsDNA	62.18	54	42961	0.624675325	Pa_Phikmv_24
NC_012418.1	Phikmvvirus	dsDNA	62.87	53	43152	0.707792208	Pa_Phikmv_25
NC_017865.1	Phikmvvirus	dsDNA	62.27	51	42966	0.594205794	Pa_Phikmv_26
NC_022091.1	Phikmvvirus	dsDNA	62.14	53	42874	0.592107892	Pa_Phikmv_27
NC_022746.1	Phikmvvirus	dsDNA	62.28	50	42957	0.596903097	Pa_Phikmv_28
NC_022971.1	Bruynoghevirus	dsDNA	52.33	69	45344	0.592307692	Pa_Bruynoghe_3
NC_027375.1	Phikmvvirus	dsDNA	62.44	54	43227	0.594305694	Pa_Phikmv_29
NC_028836.1	Phikmvvirus	dsDNA	62.2	55	42508	0.59000999	Pa_Phikmv_30
NC_047852.1	Phikmvvirus	dsDNA	62.26	49	42351	0.691008991	Pa_Phikmv_31
NC_047967.1	Phikmvvirus	dsDNA	62.19	49	42750	0.602797203	Pa_Phikmv_32

OK539824.1	Phikmvvirus	dsDNA	62.25	56	43291	0.634865135	Pa_Phikmv_33
OP292288.1	Phikmvvirus	dsDNA	62.34	54	42721	0.581418581	Pa_Phikmv_34

(g) Lytic phage genomes infecting *Staphylococcus aureus*.

Phage ID	Phage type	DNA type	GC content (%)	Number of protein	Sequence length	Class probability	Phage name
MK417514.1	Kayvirus	dsDNA	30.31	279	146004	0.729270729	Sa_Kay_1
MN047438.1	Kayvirus	dsDNA	30.24	233	148511	0.69030969	Sa_Kay_2
NC_004678.1	Rosenblumvirus	dsDNA	29.62	62	16784	0.725574426	Sa_Rosenblum_1
NC_007046.1	Rosenblumvirus	dsDNA	29.29	65	18199	0.730969031	Sa_Rosenblum_2
NC_047720.1	Kayvirus	dsDNA	30.42	109	138339	0.697702298	Sa_Kay_3

(h) Lytic phage genomes infecting *Streptococcus pneumoniae*.

Phage ID	Phage type	DNA type	GC content (%)	Number of protein	Sequence length	Class probability	Phage name
KJ617393.1	Cepunavirus	dsDNA	38.82	27	19347	0.601598402	Sp_Cepuna_1
NC_001825.1	Cepunavirus	dsDNA	38.83	25	19343	0.622177822	Sp_Cepuna_2

(i) Lytic phage genomes infecting *Salmonellae enterica*.

Phage ID	Phage type	DNA type	GC content (%)	Number of protein	Sequence length	Class probability	Phage name
EU547803.1	Teetrevirus	dsDNA	50.86	55	38815	0.71958042	Se_Teetre_1
FR667955.1	Teseptimavirus	dsDNA	48.93	47	38368	0.613786214	Se_Teseptima_1
HQ331142.1	Gelderlandvirus	dsDNA	36.88	268	160221	0.619480519	Se_Gelderland_1
KJ000058.2	Gelderlandvirus	dsDNA	36.86	259	159914	0.634265734	Se_Gelderland_2
KM366096.1	Berlinvirus	dsDNA	48.95	47	39696	0.678721279	Se_Berlin_1
KU867307.1	Gelderlandvirus	dsDNA	37.15	259	159878	0.62027972	Se_Gelderland_3
MF001354.1	Tequatrovirus	dsDNA	35.28	269	169805	0.748851149	Se_Tequatro_1
MF957259.1	Gelderlandvirus	dsDNA	36.95	264	159323	0.637662338	Se_Gelderland_4
MG471392.1	Berlinvirus	dsDNA	48.72	49	39688	0.677322677	Se_Berlin_2
MH382198.1	Teseptimavirus	dsDNA	49.25	48	38821	0.671528472	Se_Teseptima_2

MK907285.1	Berlinvirus	dsDNA	49.09	48	39176	0.613586414	Se_Berlin_3
MN026740.1	Teseptimavirus	dsDNA	48.91	46	38554	0.671528472	Se_Teseptima_3
MN252582.1	Berlinvirus	dsDNA	49.08	43	39050	0.66033966	Se_Berlin_4
MN580668.1	Tequatrovirus	dsDNA	35.36	276	172360	0.773626374	Se_Tequatro_2
MW149274.1	Dhakavirus	dsDNA	39.67	279	172418	0.669230769	Se_Dhaka_1
MZ150757.1	Tequatrovirus	dsDNA	35.3	270	169920	0.779220779	Se_Tequatro_3
NC_004831.2	Zindervirus	dsDNA	47.23	52	43769	0.65984016	Se_Zinder_1
NC_010807.1	Teetrevirus	dsDNA	50.86	55	38815	0.766733267	Se_Teetre_2
NC_015271.1	Teseptimavirus	dsDNA	48.93	47	38368	0.613786214	Se_Teseptima_4
NC_020416.1	Gelderlandvirus	dsDNA	36.88	268	160221	0.655544456	Se_Gelderland_5
NC_026607.2	Gelderlandvirus	dsDNA	36.86	259	159914	0.622277722	Se_Gelderland_6
NC_031065.1	Gelderlandvirus	dsDNA	37.15	259	159878	0.628971029	Se_Gelderland_7
NC_031258.1	Berlinvirus	dsDNA	48.95	47	39696	0.671428571	Se_Berlin_5
NC_042044.1	Gelderlandvirus	dsDNA	36.95	264	159323	0.636663337	Se_Gelderland_8
NC_048004.1	Teseptimavirus	dsDNA	49.25	49	38821	0.674725275	Se_Teseptima_5
NC_048105.1	Berlinvirus	dsDNA	48.72	49	39688	0.680619381	Se_Berlin_6
NC_054936.1	Tequatrovirus	dsDNA	35.28	269	169805	0.809490509	Se_Tequatro_4
NC_054937.1	Tequatrovirus	dsDNA	35.36	276	172360	0.794605395	Se_Tequatro_5
OL800603.1	Berlinvirus	dsDNA	48.42	47	39433	0.672827173	Se_Berlin_7
OL800605.1	Teetrevirus	dsDNA	50.42	47	38505	0.658941059	Se_Teetre_3
OM912978.1	Gelderlandvirus	dsDNA	36.95	252	159658	0.636863137	Se_Gelderland_9
ON720975.1	Tequatrovirus	dsDNA	35.48	265	169242	0.782417582	Se_Tequatro_6

(j) Lytic phage genomes infecting *Shigella flexneri*.

**Phage ID**	**Phage type**	**DNA type**	**GC content (%)**	**Number of protein**	**Sequence length**	**Class Probability**	**Genome GC/AT ratio**
AF021347.1	Myoviridae	dsDNA	39.11	49	4002	0.511388611	0.64
AF056939.1	Siphoviridae	dsDNA	41.59	288	1385	0.509390609	0.71

HM035024.1	Tunavirus	dsDNA	45.41	134	50661	0.525174825	0.83
HM035025.1	Tequatrovirus	dsDNA	35.57	136	165919	0.794105894	0.55
KC710998.1	Hanrivervirus	dsDNA	44.02	271	51821	0.512587413	0.79
KM407600.1	Mosigvirus	dsDNA	37.56	271	169062	0.684915085	0.6
KP085586.1	Tunavirus	dsDNA	45.44	272	50109	0.530669331	0.83
LC465543.1	Tequatrovirus	dsDNA	35.29	49	168000	0.770829171	0.55
MF158038.1	Cedarrivervirus	dsDNA	45.99	80	46454	0.504995005	0.85
MF158039.1	Eastlansingvirus	dsDNA	44.33	137	47647	0.537062937	0.8
MF158040.1	Mooglevirus	dsDNA	38.91	53	87570	0.502497502	0.64
MF158041.1	Mooglevirus	dsDNA	39	135	88474	0.501698302	0.64
MF158043.1	Mooglevirus	dsDNA	39.02	43	88580	0.509390609	0.64
MF158044.1	Mooglevirus	dsDNA	39	79	90270	0.508391608	0.64
MF158045.1	Tequatrovirus	dsDNA	35.51	118	166283	0.811988012	0.55
MF158046.1	Tequatrovirus	dsDNA	35.38	7	167678	0.786413586	0.55
MF327003.1	Mooglevirus	dsDNA	39.06	84	87575	0.504195804	0.64
MF327005.1	Mooglevirus	dsDNA	39.02	83	90375	0.532767233	0.64
MF327007.1	Tequatrovirus	dsDNA	35.45	88	166002	0.802697303	0.55
MF327008.1	Tequatrovirus	dsDNA	35.32	268	168112	0.794005994	0.55
MF327009.1	Tequatrovirus	dsDNA	35.35	241	168573	0.763336663	0.55
MG049919.1	Tunavirus	dsDNA	45.58	22	50552	0.506793207	0.84
MH464253.1	Kayfunavirus	dsDNA	52.41	86	40387	0.581718282	1.1
MH917278.1	Tunavirus	dsDNA	45.29	273	50219	0.522377622	0.83
MK562503.1	Bucovirus	dsDNA	54.08	91	43630	0.502897103	1.18
MK562505.1	Mooglevirus	dsDNA	39.07	271	88821	0.522577423	0.64
MK685668.1	Berlinvirus	dsDNA	48.4	4	40058	0.623276723	0.94
MK759854.1	Deseoctovirus	dsDNA	46.52	2	44605	0.504795205	0.87
MN296515.1	Tunavirus	dsDNA	45.35	80	50309	0.534465534	0.83
MN342247.1	Tunavirus	dsDNA	45.2	265	50411	0.558841159	0.82
MN432485.1	Hanrivervirus	dsDNA	44.1	65	51296	0.515184815	0.79

MN781580.1	Tequatrovirus	dsDNA	35.56	94	167485	0.754345654	0.55
MW341595.1	Tequatrovirus	dsDNA	35.62	54	164878	0.752347652	0.55
MW822011.1	Tequatrovirus	dsDNA	35.43	79	169805	0.505994006	0.55
MZ358387.1	Schitoviridae	dsDNA	44.64	66	72458	0.505394605	0.81
NC_015456.1	Tunavirus	dsDNA	45.41	80	50661	0.537162837	0.83
NC_015457.1	Tequatrovirus	dsDNA	35.57	265	165919	0.797702298	0.55
NC_021331.1	Hanrivervirus	dsDNA	44.02	94	51821	0.517482517	0.79
NC_025437.1	Mosigvirus	dsDNA	37.56	267	169062	0.741358641	0.6
NC_026010.1	Tunavirus	dsDNA	45.44	83	50109	0.552447552	0.83
NC_042039.1	Tequatrovirus	dsDNA	35.51	267	166283	0.8	0.55
NC_042077.1	Tequatrovirus	dsDNA	35.45	266	166002	0.804295704	0.55
NC_042078.1	Tequatrovirus	dsDNA	35.32	271	168112	0.775724276	0.55
NC_047929.1	Drulisvirus	dsDNA	50.94	49	43036	0.51038961	1.04
NC_048025.1	Kayfunavirus	dsDNA	52.41	49	40387	0.576623377	1.1
NC_048174.1	Bucovirus	dsDNA	54.08	53	43630	0.514285714	1.18
NC_048180.1	Berlinvirus	dsDNA	48.4	43	40058	0.660839161	0.94
NC_049831.1	Tunavirus	dsDNA	45.35	84	50309	0.526873127	0.83
NC_054941.1	Tequatrovirus	dsDNA	35.38	271	167678	0.773426573	0.55
OK018184.1	Tequatrovirus	dsDNA	35.59	267	168362	0.804195804	0.55
OP197928.1	Tequatrovirus	dsDNA	35.59	83	167011	0.797502498	0.55

Table S3: List of inhibitor proteins from the lytic ESKAPE phages.

(a) Lytic phage genomes infecting *Escherichia coli*.

Phage name	Phage ID	Protein ID	Protein function	Sequence length	Sequence
Tequatrovirus T4	P32267	P32267.1	anti-sigma factor	90	MNKNIDTVREIITVASILIKFSREDIVENRANFIAFLNEIGVTHEGRKLNQNSFRKIVSELTQEDKKTLIDEFNEGFEGVYRYLEMYTNK
Escherichia phage T4	MT984581.1	QPI17481.1	inhibitor of MrcBC restriction endonuclease	94	MIIDSQSVVQYTFKIDILEKLYKFLPNLYHSIVNELVEELHLENNDFLIGTYKDLSKAGYFYVIPAPGKNIDDVLKTIMIYVHDYELKIISNES
Escherichia phage vB_EcoM_Nami	MZ502380.1	QXV73373.1	RNase III inhibitor	159	MIVKYIKGDIVALFLQGNIIAHGCNCFHTMGSGVAGQLARAYPKILEIDKTTTEYGSRDKLGDMSIVFKHNPTGFGICYNLYTQYEPGPNLDYGALVNCMIELNLQAETLLFKPVIYIPRIGCGIAGGDWDKVSKLIDMFTPDIDLIVVDYESTLPASV
Escherichia phage Paracelsus	MZ501096.1	QXV83130.1	type IC restriction inhibitor	28	MSNFHNEHVMQFYRNNLKNFGILGSKNS
Escherichia phage vB_EcoM-G28	NC_054916.1	YP_010069702.1	internal virion protein	96	MKTFKEFTSTTTTPVSTITEATLTSEVIKANKGREGKPMISLVDGEEIKGTVYLGDGWSAKKDGATIVISPAEETALFKAKHISAAHLKIIAKNLL

(b) Lytic phage genomes infecting *Staphylococcus aureus*.

Phage name	Phage ID	Protein ID	Protein function	Sequence length	Sequence

Staphylococcus phage vB_SauM-515A1	MN047438.1	QEA03056.1	DNA sliding clump inhibitor	58	MVIPSIKAQNKFKNELEYYKQGHISESKMLELAFDYIQELEQNNEYVTNLLEEERYGE

(c) Lytic phage genomes infecting *Salmonellae enterica*.

**Phage name**	**Phage ID**	**Protein ID**	**Protein function**	**Sequence length**	**Sequence**
Salmonella phage vB_SnwM_CGG4-1	KU867307.1	ANA49419.1	host protease inhibitor	144	MSLVNKVFEIIEDDEELLEQFPEMKVGTVFKVITVDKENGHEDGITSVKIKNGPYLHINARDSWFWCFYCDDGMYQLKEIEEHDSDLFPAVTLGFFDGQLITDHLEAAKIRVNGYEANIMQAAADYIRALEKQLSFSGEDLNET
Salmonella phage SG1	MF001354.1	ASZ76185.1	lysis inhibitor	312	MYNIKCLTKNEQAEIVKLYSSGNYTQQELADWQGVSVDTIRRVLKNAEEAKHPKVTISGGITVKVNSDAVIAPVAKSDIIWNASKKFISITVDGVTYNATPNTHSNFQEILNLLVADKLEEAAQKINVRRAVEKYISGDVRIEGGSLFYQNIELRSGLVDRILDSMEKGENFEFYFPFLENLLENPSQKAVSRLFDFLVANDIEITEDGYFYAWKVVRSNYFDCHSNTFDNSPGKVVKMPRTRVNDDDTQTCSRGLHVCSKSYIRHFSSSTSRVVKVKVHPRDVVSIPIDYNDAKMRTCQYEVVEDVTEQFK
Salmonella phage SG1	MF001354.1	ASZ76263.1	protease inhibitor	161	MITVDKWFRINRVDTGLCNYWPELSAGTVFKVRELAKECEDDIEPDTGIIEIELSDGKIINIYDKPITYWCLWNTESVENGEIEEVVERTSQDVQKPKAAFQGERISYALAKLAAQENND

					GYEGNLMQAAAEYIEWLETQISFSDQKIRQYKRLNQMFYNT
Salmonella phage LPST144	MN252582.1	QEP53483.1	deoxyguanosine triphosphohydrolase inhibitor	86	MGRLYSGSLAAYKSAIESLEVLGCNVKVKHEDIVSRRVRAIHIEVSMMTSGDMMGKETFMYYDDDVLFNTCTAWMRKLAEEIRSWK
Salmonella phage C2	MN026740.1	QGK90534.1	inhibitor of recBCD nuclease	52	MSRDLVTIPRDVWNDMQGYIDSLERENDSLKNQLMEADEYVAELEEKLNGAS
Salmonella phage vB_STy-RN5i1	OL800603.1	UJD21320.1	inhibitor of host toxin/antitoxin system	100	MAIKFPGNTIRLSDTIDQYSRRVHINVRNGKVTLVYRWKDHKSTKSHTQRVTLDDTQAGRLLASVAVAATVAVGEDKTRELLLSKVAGEEGMVLSDKSGI
Salmonella phage vB_STy-RN5i1	OL800603.1	UJD21325.1	dGTP triphosphohydrolase inhibitor	90	MGRLYSGNLIAYKDAIERLKEDHDANVVVETYRYEDFTQRRMVAGETLRVVLRDGLTLTAKCFEQSDEDVRCNAQTEWLRKVHSDMKHWK
Salmonella phage vB_SAg-RPN15	OL800605.1	UJD21528.1	dGTP triphosphohydrolase inhibitor	87	MMMGRLYSGNLNDFKAATNKLFGLDLAVICDDLYEDNWHIQGLRVSVEDRTGNLIDSRTFFHRDEDVLYIMVTAWLNHMYDQLKDWK
Salmonella phage vB_SAg-RPN15	OL800605.1	UJD21536.1	host recBCD nuclease inhibitor	60	MIMPKSDTVTMTRDAWNDVSAYIDKLEKDLEFLNALKACGVDNWDGYSDAVEMVYGEDDE
Salmonella phage vB_SAg-RPN15	OL800605.1	UJD21538.1	inhibitor of host bacterial RNA polymerase	54	MEQREQKYLLTIEGNTESFEVPVFARSLEEATLQAEHYEDAGFVVTRIRPEVKA
Salmonella phage Vi06	NC_015271.1	YP_004306663.1	RNA polymerase inhibitor	80	MVYPRATTWSFTINGYREVTDMLNVGTDSKKFWATVESSEHSFEVPVYAETLDEALEL

					AEWQYVQAGFEVTRVRPCVTPK
Salmonella phage SG1	NC_054936.1	YP_010075074.1	minor head protein inhibitor of protease	226	MIDKDYIAELKALDDNKEAKAKLAEYAEQFGIKVKKNKSFDNIVNDIEEALQKLASEPMPETDGLSIKDLINAADAAEGLKYDDEEVNPEAALLIDSPVKSDIKIEVVETDKIPENTDVLIEDTPFVEEKFEQAVAEIIESEKPSVFTLPENFSPNLQLIGKNPGFCTVPWWIYQWIAETPDWKSHPTSFEHASAHQTLFSLIYYINRDGSVLIRETRNSSFVTLK
Salmonella phage SG1	NC_054936.1	YP_010075102.1	lysis inhibition; accessory protein	82	MIKQLQHALELQRNAWNNGHENYGASIDVEAEALEILRYFKHLNPAQTALAAELQEKDELKYAKPLASAARKAVRHFVVTLK
Salmonella phage SG1	NC_054936.1	YP_010075166.1	rIIA lysis inhibitor	725	MIITTEKETILGNGSKSKAFSITASPKVFKILSSDLYTNKIRAVVRELITNMIDAHALNGNPEKFIIQVPGRLDPRFVCRDFGPGMSDFDIQGDDNSPGLYNSYFSSSKAESNDFIGGFGLGSKSPFSYTDTFSITSYHKGEIRGYVAYMDGDGPQIKPTFVKEMGPNDKTGIEIVVPVEEKDFRNFAYEVSYIMRPFKDLAIINGLDREIDYFPDFDDYYGVNPERYWPDRGGLYAIYGGIVYPIDGVIRDRNWLSIRNEVNYIKFPMGSLDIAPSREALSLDDRTRKNIIERVKELSEKAFNEDVKRFKESTSPRHTYRELMKMGYSARDYMISNSVKFTTKNLSYKKMQSMFEPDSKLCNAGVVYEVNLDPRLKRIKQSHETSAVASSYRLFGINTTKINIVIDDIKNRVNIVRGLAHALDDSEFNNTLNIHHNERLLFI

					NPEVESQIDLLPDIMAMFES<br>DEVNIHYLSEIEALVKSYIPK<br>VVKSKAPRPKAATAFKFEIK<br>DGRWEKEELFTLTSEADEIT<br>GYVAYMHRSDIFSMDGTTS<br>LCHPSMNILIRMANLIGINEF<br>YVIRPLLQKKVKELGQCQCI<br>FEALRDLYVDAFDDVDYDK<br>YVGYSSSAKRYIDKIIKYPEL<br>DFMMKYFSVDEVSEEYTRL<br>ANMVSSLQGVYFNGGKDTI<br>GHDIWTVTNLFDVLSNNAS<br>KNSDKMVAEFTKKFRIVSDF<br>IGYRNSLSDDEVSQIAKTMK<br>ALAA
Salmonella phage SG1	NC_054936.1	YP_010075167.1	rIIA lysis inhibitor	67	MKSYKVNLELFDKAVHREY<br>RIIQRFFDMGEAEEFKNRFK<br>DIRDKIQSDTATKDELLEVAE<br>VIKRNMN
Salmonella phage pSe_SNUABM_01	NC_054937.1	YP_010075492.1	inhibitor of host transcription	167	MDLQLITTEMVVEAYGDTTD<br>GISVFKGNRRVGYITDLKKD<br>LAKQVKRKTTIKEYRNRRLE<br>QARDMLPDAVEEMKVFLEN<br>QLAKYDCDVFINQTQPNVHI<br>NNCKCYIIVNPLTGKHRLGIS<br>NPNRSASDMAEDVEACFKI<br>SKSPAEHHILINGLSQDDIIE<br>VIKTLCM
Salmonella phage pSe_SNUABM_01	NC_054937.1	YP_010075524.1	inhibitor of MrcBC restriction	92	MIIDSQSVVQYTIKIDILEKLY<br>KFLPNLYHSIVNELVEELHLE<br>NNDFLVGTYKDNSKAGYFYI<br>IPAPGKSIDDVLKTIMIYVHD<br>YEIEDYFE
